# Pangenome Analytics Reveal Two-Component Systems as Conserved Targets in ESKAPEE Pathogens

**DOI:** 10.1128/mSystems.00981-20

**Published:** 2021-01-26

**Authors:** Akanksha Rajput, Yara Seif, Kumari Sonal Choudhary, Christopher Dalldorf, Saugat Poudel, Jonathan M. Monk, Bernhard O. Palsson

**Affiliations:** a Systems Biology Research Group, Department of Bioengineering, University of California, San Diego, San Diego, California, USA; b Bioinformatics and Systems Biology Program, University of California, San Diego, San Diego, California, USA; c Department of Pediatrics, University of California, San Diego, San Diego, California, USA; d Novo Nordisk Foundation Center for Biosustainability, Technical University of Denmark, Kemitorvet, Kongens, Lyngby, Denmark; University of Vienna

**Keywords:** ESKAPEE pathogens, pangenomic analysis, two-component systems, antibiotic resistance, genomic architecture

## Abstract

The two-component system (TCS) helps bacteria sense and respond to environmental stimuli through histidine kinases and response regulators. TCSs are the largest family of multistep signal transduction processes, and they are involved in many important cellular processes such as antibiotic resistance, pathogenicity, quorum sensing, osmotic stress, and biofilms. Here, we perform the first comprehensive study to highlight the role of TCSs as potential drug targets against ESKAPEE (Enterococcus faecium, Staphylococcus aureus, Klebsiella pneumoniae, Acinetobacter baumannii, Pseudomonas aeruginosa, *Enterobacter* spp., and Escherichia coli) pathogens through annotation, mapping, pangenomic status, gene orientation, and sequence variation analysis. The distribution of the TCSs is group specific with regard to Gram-positive and Gram-negative bacteria, except for KdpDE. The TCSs among ESKAPEE pathogens form closed pangenomes, except for Pseudomonas aeruginosa. Furthermore, their conserved nature due to closed pangenomes might make them good drug targets. Fitness score analysis suggests that any mutation in some TCSs such as BaeSR, ArcBA, EvgSA, and AtoSC, etc., might be lethal to the cell. Taken together, the results of this pangenomic assessment of TCSs reveal a range of strategies deployed by the ESKAPEE pathogens to manifest pathogenicity and antibiotic resistance. This study further suggests that the conserved features of TCSs might make them an attractive group of potential targets with which to address antibiotic resistance.

**IMPORTANCE** The ESKAPEE pathogens are the leading cause of health care-associated infections worldwide. Two-component systems (TCSs) can be used as effective targets against pathogenic bacteria since they are ubiquitous and manage various vital functions such as antibiotic resistance, virulence, biofilms, quorum sensing, and pH balance, among others. This study provides a comprehensive overview of the pangenomic status of the TCSs among ESKAPEE pathogens. The annotation and pangenomic analysis of TCSs show that they are significantly distributed and conserved among the pathogens, as most of them form closed pangenomes. Furthermore, our analysis also reveals that the removal of the TCSs significantly affects the fitness of the cell. Hence, they may be used as promising drug targets against bacteria.

## Abstract (precis)

The ESKAPEE pathogens are the leading cause of health care-associated infections worldwide. Two-component systems (TCSs) can be used as effective targets against pathogenic bacteria since they are ubiquitous and manage various vital functions such as antibiotic resistance, virulence, biofilms, quorum sensing, and pH balance, among others.

## INTRODUCTION

Two-component systems (TCSs) are ubiquitous among bacterial species (1, 2). They participate in numerous cellular processes, including signaling and pathogenicity (3), and also play a major role in the pathogenicity of the highly infectious ESKAPEE group of pathogens, which is an acronym for Enterococcus faecium, Staphylococcus aureus, Klebsiella pneumoniae, Acinetobacter baumannii, Pseudomonas aeruginosa, *Enterobacter* spp., and Escherichia coli (4, 5). The ESKAPEE pathogens, consisting of both Gram-positive and Gram-negative bacteria, are the leading cause of nosocomial life-threatening infections and are in the WHO’s “priority pathogen” list (6). The problem of trying to tackle nosocomial infection worsens due to the increase in antibiotic resistance and virulence.

**FIG 1:**
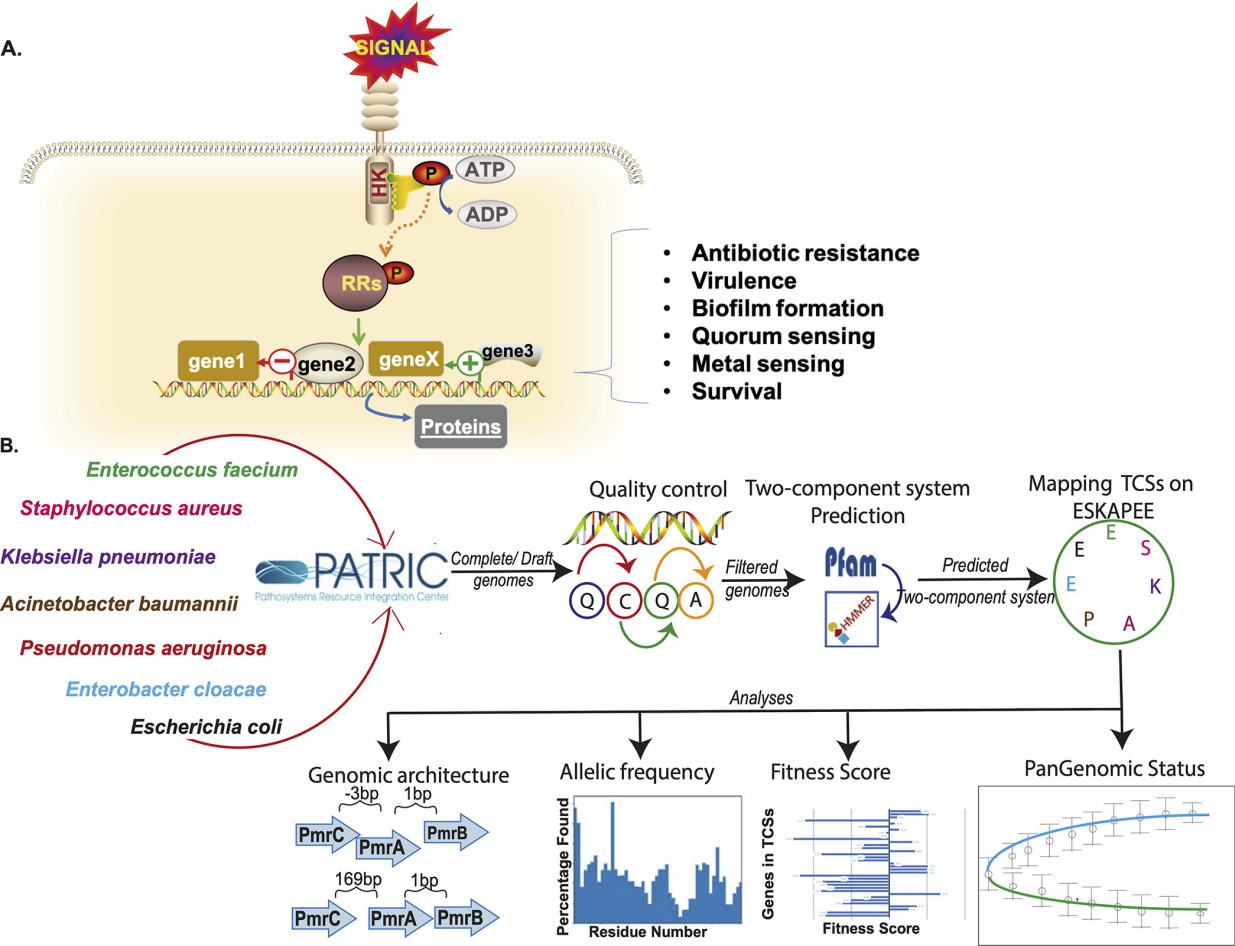
Pangenome analysis of two-component systems. (A) Schematic diagram depicting the mechanism of a two-component system. (B) Flow chart showing the methodology used in the study.

The histidine kinase (HK) and response regulator (RR) are two important components of TCSs (7). HKs are typically transmembrane proteins that sense external signals; however, in a few instances, they are cytoplasmic (8–10). In general, stimulus detection led to conformational changes that further affected the autokinase activity of the C-terminal kinase core; the phosphoryl group was transferred onto the aspartate residue of the cognate RR. Furthermore, the phosphorylated RR mediates the activity of the associated effector domain of the response regulator protein, which further modulates appropriate responses (11–13). Besides the autokinase activity, many HKs exhibit a phosphatase activity toward the cognate phosphorylated RRs, e.g., CheA/Z and KdpD, etc. (14, 15) (Fig. 1A). However, the RRs do not always modulate the downstream responses by transcription, as a significant number of them do not affect transcription (16). Thus, TCSs help bacteria acclimatize to a wide range of external factors.

TCSs are involved in antibiotic resistance, virulence, quorum sensing, biofilm formation, metal sensing, motility, survival, and many other functions (8, 17). The antibiotic resistance TCSs help bacteria address the presence of various antibiotics (18). The TCSs involved in virulence help sustain bacteria in the host or at the site of pathogenicity (19). The quorum sensing-, motility-, and biofilm-related TCSs allow bacteria to communicate, move, and form colonies to acclimatize to unfavorable environments (19, 20). Furthermore, bacteria also have TCSs to tackle various conditions such as high pH, metals, anaerobic conditions, and nutrient sensing, etc. (8, 21). Therefore, the many roles played by TCSs make them a valuable potential target for antimicrobials. Several studies have confirmed this potential (22, 23).

Among all the functions of TCSs, antibiotic resistance is important among the nosocomial-infection-causing ESKAPEE group of pathogens (6, 18). Bacteria adapt different TCS mechanisms to express antibiotic resistance phenotypes (24). The mechanisms include overexpression of efflux pumps, cell surface modifications, upregulation of antibiotic resistance genes, and increased biofilm formation (18, 25). Various strategies need to be developed to overcome these specialized modifications against antibiotics in bacteria.

TCSs are a fundamental determinant of bacterial physiological states. Despite being ubiquitous and vital for bacterial survival, TCSs have not yet been the subject of a detailed pangenomic analysis. A pangenomic study would be helpful to understand the conservation status of all the TCSs involved in antibiotic resistance, virulence, biofilm, and motility and others involved in the basic survival mechanisms in bacteria. The literature shows that TCSs could be a promising target to fight the pathogenicity of bacteria, especially antibiotic resistance (26). This pangenome study, driven by the availability of a large number of strain-specific genome sequences, is focused on exploring all TCSs and determining them as potential targets against the ESKAPEE pathogens.

## RESULTS

### Annotation of two-component systems.

**FIG 2:**
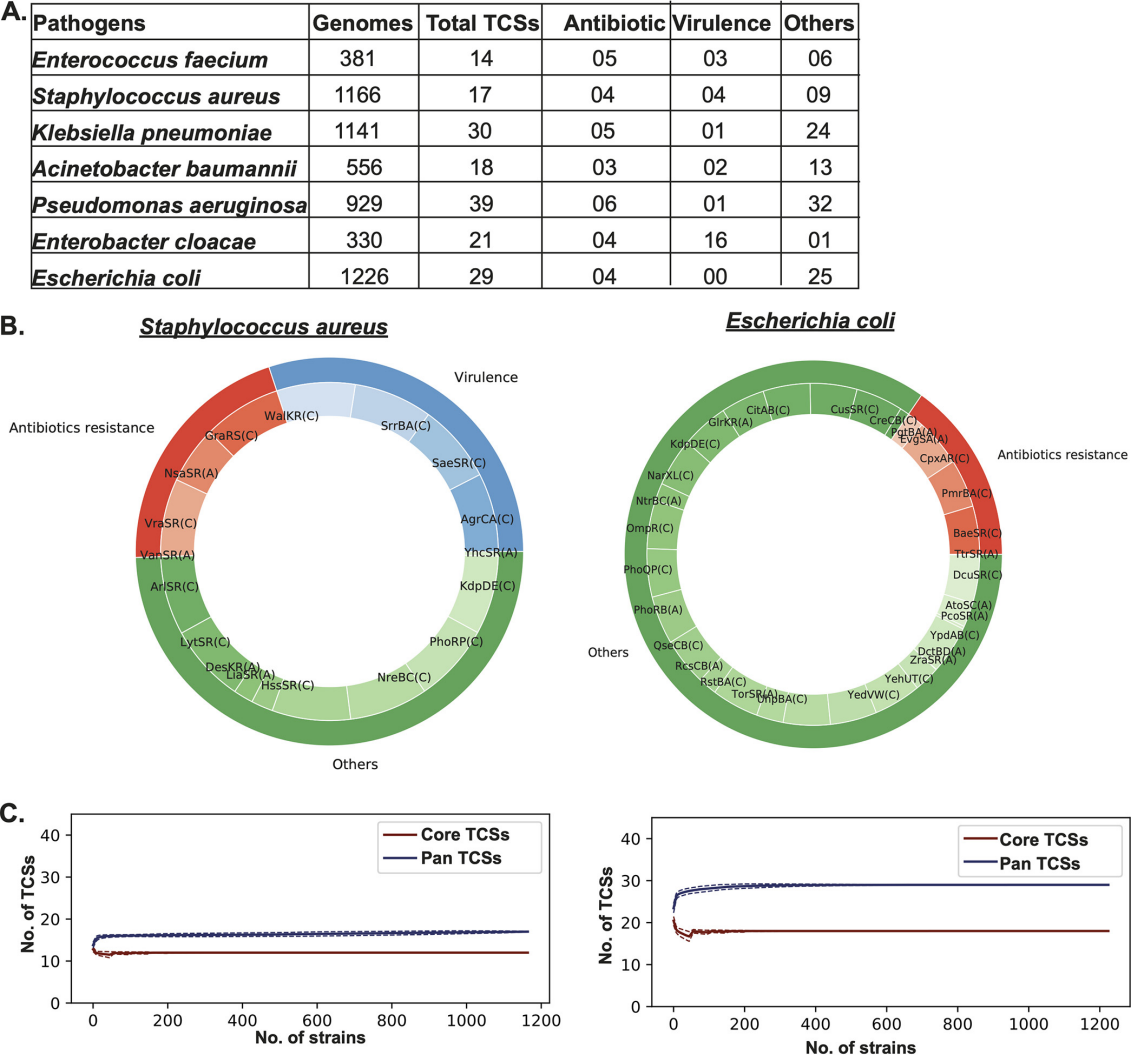
Annotated two-component systems (TCSs) among the ESKAPEE pathogens. The TCSs were predicted and annotated using an hmmsearch of the respective Pfam profiles of the histidine kinases and response regulators. (A) Table showing the total number of genomes after quality control (QC) and TCSs annotated and categorized as antibiotic resistance, virulence, and others. The draft/complete genomes were downloaded from the PATRIC database. QC involves steps such as multilocus sequence typing (MLST), <100 contigs, coding sequences (CDSs) between the [average ± 2(standard deviation)], and <1,000 N’s in genomes. The Pfam profiles of each TCS were extracted using the hmmsearch approach. Furthermore, the TCSs were categorized into different categories according to their function reported in the literature, e.g., antibiotics resistance, virulence, and others (quorum sensing, biofilm, motility, and sporulation, etc.). (B) Multilevel pie chart depicting the distribution of TCSs in different functional categories among S. aureus and E. coli strains. The multilevel pie chart is divided into two concentric circles: the outer circle represents the function of TCSs, such as antibiotic resistance (red), virulence (blue), and others (green), while the inner circle represents the TCSs falling into specific categories along with their distribution frequencies, i.e., core (C), accessory (A), and unique (U). The core pangenomic status includes the TCSs found in >98% of strains, and accessory includes the TCSs found in between 1 and 98% of strains, while the unique status of TCSs represents the TCSs found in only 1 strain. (C) Pangenome curves for S. aureus and E. coli. The curves show the conservation statuses of core and pan-TCSs. The plot is constructed between the number of TCSs and the number of strains. In the case of S. aureus and E. coli, the graph shows that both the core and pan-TCSs remain constant with the increase in the number of strains.

Different numbers of TCSs were annotated among ESKAPEE pathogens using the hidden Markov model (HMM) approach (Fig. 2B). We categorized the TCSs into four different groups, namely, antibiotic resistance, virulence, others (general), and predicted family. We put the TCSs associated with pH, motility, quorum sensing, and biofilms, etc., in the “others” category because for this article, we are interested in those functions that have a higher priority in antibiotic research, such as antibiotic resistance and virulence. Additionally, the “predicted family” includes the TCSs whose family has been annotated rather than the exact TCS. A detailed list of TCSs and their functions among ESKAPEE pathogens is provided in Data Set S1, sheet 1, in the supplemental material.

10.1128/mSystems.00981-20.10 DATA SET S1 (Sheet 1) Detailed list of two-component systems and their functions among Enterococcus faecium, Staphylococcus aureus, Klebsiella pneumoniae, Acinetobacter baumannii, Pseudomonas aeruginosa, Enterobacter cloacae, and Escherichia coli (ESKAPEE). (Sheet 2) Detailed list of the two-component system genes with essential functions along with the organisms, identifiers, and pangenome statuses. (Sheet 3) Fitness scores of the TCS genes of Acinetobacter baumannii and Klebsiella pneumoniae. (Sheet 4) Table showing the Pfam profiles used to extract the two-component systems. Download Data Set S1, XLSX file, 1.1 MB. Copyright © 2021 Rajput et al. 2021 Rajput et al. This content is distributed under the terms of the Creative Commons Attribution 4.0 International license.

The highest number of TCSs, i.e., 39, were mapped in P. aeruginosa, with 6 functioning in antibiotic resistance and 1 functioning in virulence, with the remaining 32 falling into the other (general) category. Among ESKAPEE pathogens, E. faecium has 14 TCSs, which is the lowest in number, with 5 functioning in antibiotic resistance. Other ESKAPEE pathogens such as K. pneumoniae, E. coli, Enterobacter cloacae, A. baumannii, and S. aureus mapped with 30, 29, 21, 18, and 17 TCSs, respectively (Fig. 2A). The highest number of TCSs involved in antibiotic resistance is present in P. aeruginosa, while the highest number of TCSs for virulence is found in E. cloacae. The TCSs with other (general) functions are most abundant in P. aeruginosa.

### Pangenome analysis of two-component systems.

The pangenome analysis of the TCSs among the ESKAPEE pathogens showed that most of the TCSs are part of the “accessory” and “core” pangenomes; i.e., they are shared across the genome (Fig. 2B and Fig. S4). The percentages of core, accessory, and unique pangenomes are 45.24%, 50.60%, and 4.17%, respectively. The conservation status of the TCSs is also depicted as a pangenome curve showing core and pangenome TCSs (Fig. 2C and Fig. S5).

10.1128/mSystems.00981-20.4 FIG S4 Pangenome analysis of the two-component systems in the form of a multilevel pie chart depicting the distribution of TCSs in all four categories in Enterococcus faecium, Klebsiella pneumoniae, Acinetobacter baumannii, Enterobacter cloacae, and Pseudomonas aeruginosa. Download FIG S4, PDF file, 0.1 MB. Copyright © 2021 Rajput et al. 2021 Rajput et al. This content is distributed under the terms of the Creative Commons Attribution 4.0 International license.

10.1128/mSystems.00981-20.5 FIG S5 Pangenome analysis of the two-component systems in the form of a pangenome curve for Enterococcus faecium, Klebsiella pneumoniae, Acinetobacter baumannii, Enterobacter cloacae, and Pseudomonas aeruginosa. The curve shows the statuses of the core genome and pangenome for TCSs. All the pathogens show a “closed” pangenome for TCSs except for P. aeruginosa. Download FIG S5, PDF file, 0.1 MB. Copyright © 2021 Rajput et al. 2021 Rajput et al. This content is distributed under the terms of the Creative Commons Attribution 4.0 International license.

**FIG 3:**
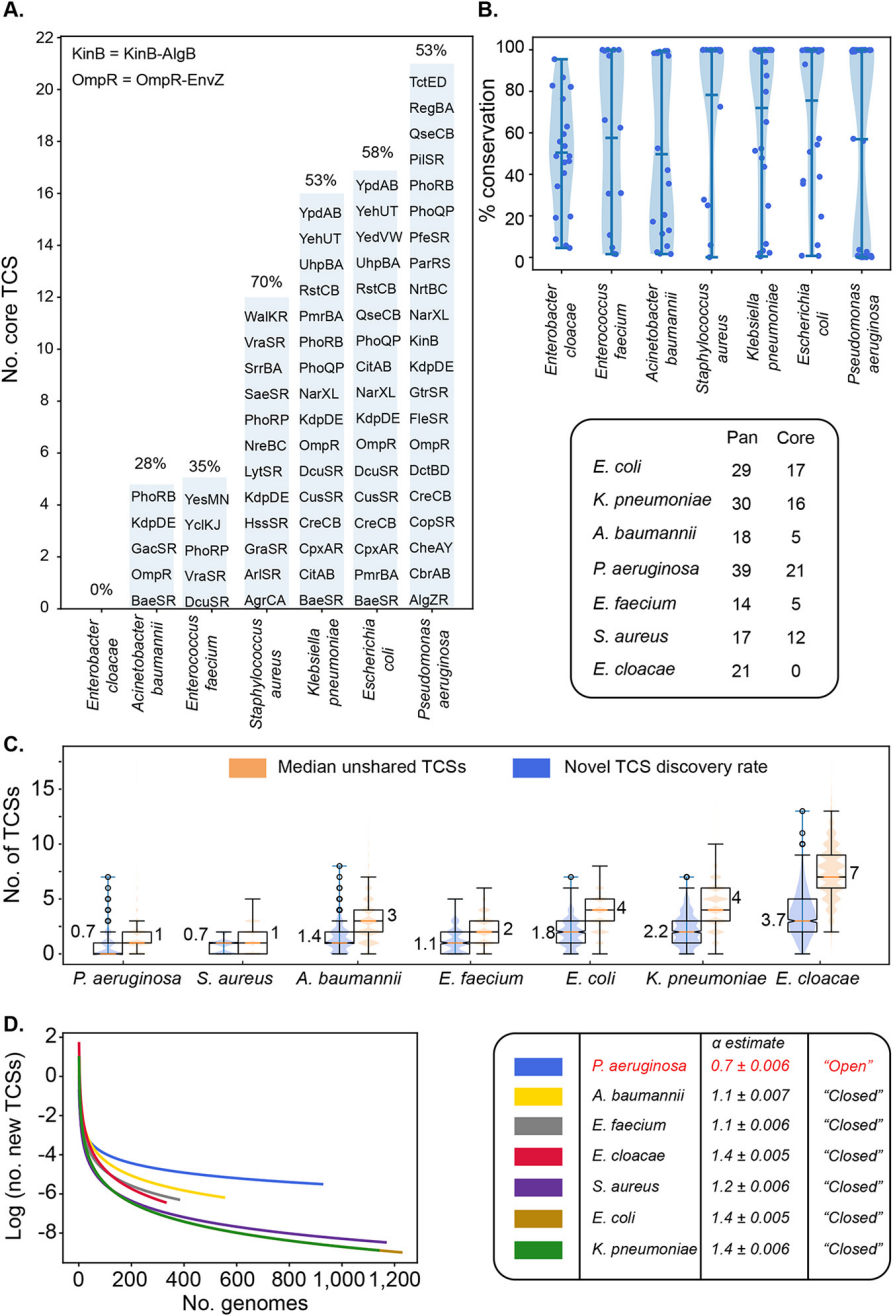
Pan status of two-component systems (TCSs) among ESKAPEE pathogens. Core TCSs are defined if they are found in >98% of strains. The open and closed statuses of the TCSs are estimated by Heaps’ law. (A) Core TCSs across species. A core TCS is defined as a two-component system gene present in more than 98% of the strains. The percentage of TCSs that are part of the core is displayed at the top of each bar. (B) TCSs are variably conserved across strains. The percentage of strains in which a TCS is present is calculated for each TCS, and the distribution of percentages is plotted for each species. (C) TCS discovery curves. The number of new TCSs discovered as more strains are taken into consideration decreases across species. Heaps’ law was fitted to each curve, and the decay rate was estimated. A decay rate that is >1 indicates a closed pangenome. P. aeruginosa is the only species with a decay rate of <1, suggesting that the number of TCSs are unbounded and that new genes will constantly be discovered as new P. aeruginosa genomes are sequenced. In contrast, the set of TCSs in all six other species is bounded and ceases to increase as more strains are sequenced. (D) Median unshared TCSs and novel gene discovery rates at step 1 of the gene discovery curves in panel C. The novel TCS discovery rate represents the average number of new TCSs discovered when two strains are drawn randomly, and the gene content of the second strain is compared to that of the first strain. The median unshared TCSs represent the number of two-component systems that differ between two strains (i.e., the difference between the intersection and the union of the two sets).

**FIG 4:**
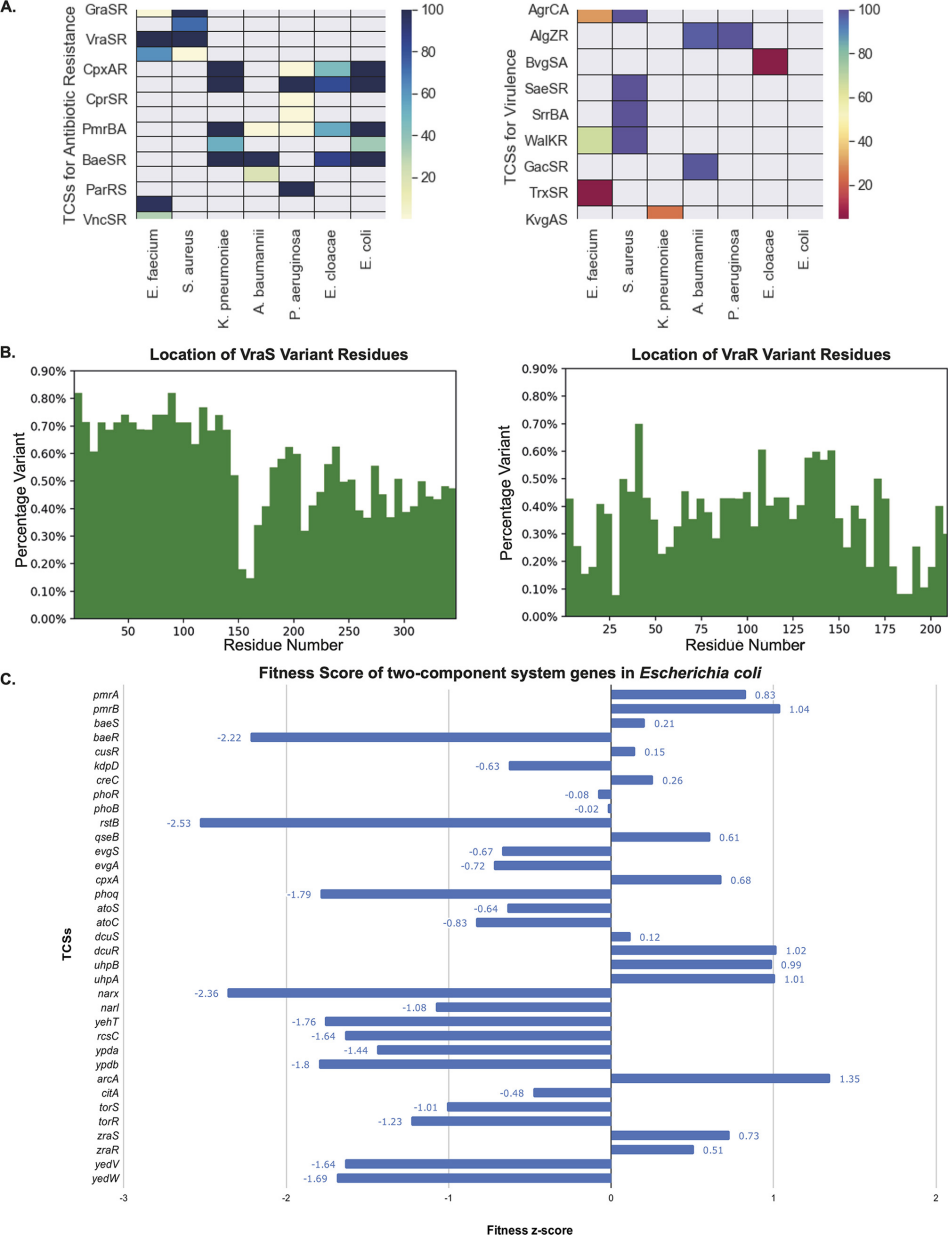
Analysis of the two-component systems (TCSs) among the ESKAPEE pathogens and variation among the histidine kinases (HKs) and response regulators (RRs). (A) Heat maps depicting the TCSs involved in antibiotic resistance and virulence. The colors of the boxes are in accordance with the distribution of TCSs in the strains of the respective ESKAPEE pathogens. The distributions of the TCSs are group specific, i.e., between Gram-positive (Staphylococcus aureus and Enterococcus faecium) and Gram-negative (Klebsiella pneumoniae, Acinetobacter baumannii, Pseudomonas aeruginosa, Enterobacter cloacae, and Escherichia coli) bacteria. (B) Sequence variant bar graphs of VraS and VraR TCSs. The graph is plotted as the percent variation versus the number of residues. The HK (VraS) shows more variation than the RR (VraR). Among the VraS TCSs, the N terminus shows more variability than the C terminus. (C) Fitness score plot of the TCS genes in Escherichia coli, plotted as the TCSs versus the fitness Z-scores. A negative fitness Z-score indicates that any mutation in the gene is more detrimental than the average mutation during infection and results in a negative effect on the pathogen.

Our first goal was to characterize the level of conservation of the two-component systems across species. We constructed core and pangenome curves focused on the TCSs for each species (see Materials and Methods). Briefly, the core genome curve corresponds to the number of conserved TCSs, and the pangenome curve reflects the total number of TCSs as more strains are taken into account. This is the first attempt to categorize TCSs into the core genome and the pangenome. Our initial categorization is focused on the following five criteria:

1. The number of TCSs found in core genomes of ESKAPEE pathogens. We find that the number of TCSs that are part of the core genome (i.e., present in more than 98% of genomes of a species (see Materials and Methods) varies across species. In total, P. aeruginosa strains have the largest number of core TCSs (*n *= 21), followed by E. coli (*n* = 17), K. pneumoniae (*n* = 16), S. aureus (*n* = 12), A. baumannii (*n* = 5), and E. faecium (*n* = 0). Surprisingly, none of the TCSs are part of the E. cloacae core genome (Fig. 3A).
2. Common TCSs among ESKAPEE pathogens. The TCSs were mapped and depicted in the form of heat maps to summarize their shared and unshared statuses along with pangenomic statuses among ESKAPEE pathogens. A summary of TCSs involved in antibiotic resistance and virulence is provided in Fig. 4A, with predicted family and others (general) in Fig. S6. Most of the TCSs are shared among the pathogens. For example, the antibiotic resistance TCS PmrBA is shared among K. pneumoniae, A. baumannii, P. aeruginosa, E. cloacae, and E. coli. A TCS involved in virulence, AlgZR, is found in A. baumannii and P. aeruginosa. The KdpDE TCS, which is involved in other (general) functions, is distributed among S. aureus, K. pneumoniae, P. aeruginosa, E. cloacae, and E. coli (Fig. 4A). However, the functions of certain core TCSs are similar across species.
3. Percentage of TCSs found in the core genomes of given ESKAPEE pathogens. While P. aeruginosa has the largest number of core TCSs, the proportion of core TCSs versus pan-TCSs is highest in S. aureus (70%). In fact, the percentage of strains sharing any one of the TCSs varies greatly within and across species, with generally high percentages of conservation in S. aureus (78%), K. pneumoniae (72%), and E. coli (75%) (Fig. 3B). In contrast, a TCS is shared in only 48%, 58%, and 50% of strains, on average, in E. cloacae, E. faecium, and A. baumannii, respectively. The distribution of percent conservation of TCSs is bimodal in P. aeruginosa.
4. Pangenomic status of TCSs for a given ESKAPEE pathogen. We investigated whether the set of TCSs was finite across a species and whether we would continue to discover new TCSs as new strains are sequenced. For this purpose, we fitted Heaps’ law to a curve plotting the number of new genes discovered as more strains are taken into account (Fig. 3C; see also Materials and Methods). Two parameters, α and *k*, are estimated when fitting Heaps’ law. When α is <1, we consider the pangenome to be “open”; i.e., we would expect to find new TCSs as more strains are sequenced indefinitely. This condition applied only to the new gene discovery curve of P. aeruginosa, revealing that the set of TCSs is finite in all of the other species.
5. TCSs shared between two strains of the same species. We plotted the average number of new TCSs discovered when a second strain is examined and the number of unshared genes between any two strains (Fig. 3D). Despite having the largest α, P. aeruginosa strains had the lowest average number of unshared TCS genes (*n* = 1) and the lowest new TCS discovery rate (0.7), while E. cloacae had the highest values for both the number of unshared TCSs (*n* = 7) and novel TCS discovery rate (3.7).

10.1128/mSystems.00981-20.6 FIG S6 Pangenome analysis of the two-component systems. (A) Heat maps depicting the TCSs involved in the other (general) category. (B) Heat maps depicting the predicted family of TCSs. Download FIG S6, PDF file, 0.1 MB. Copyright © 2021 Rajput et al. 2021 Rajput et al. This content is distributed under the terms of the Creative Commons Attribution 4.0 International license.

### Gene essentiality and fitness score.

We checked the essential genes and fitness scores of the TCSs, confirming their potential role as promising drug targets. The 9 essential genes from various TCSs, e.g., *vraS*, *walK*, *cheY*, *algR*, *kdpE*, *evgS*, *rstB*, *dcuR*, and *torR*, are shown in Data Set S1, sheet 2. To get more accurate details of the gene contribution to cell fitness, we calculated the fitness scores of the genes of TCSs (shown in Fig. 4C and Data Set S1, sheet 3). Among E. coli, K. pneumoniae, and A. baumannii, we found 31 out of the 48 genes with negative fitness Z-scores. The negative Z-scores suggest that any mutation (e.g., insertion or deletion, etc.) in the gene is more detrimental than the average mutation during infection and results in a negative effect on the pathogen.

Furthermore, the fitness scores of the genes in various TCSs could be used as promising targets to tackle the pathogenic bacteria.

### Genomic architecture of two-component systems.

**FIG 5:**
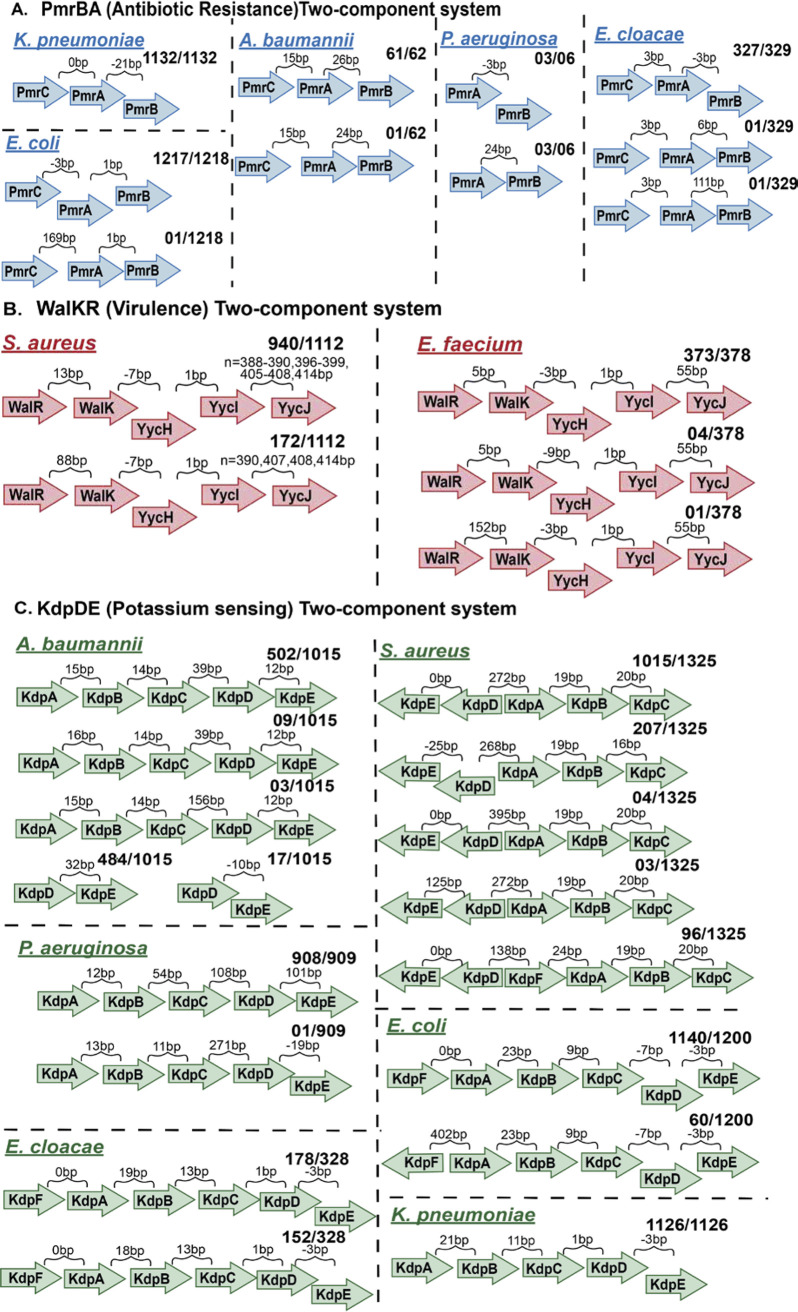
Gene orientation among the two-component systems (TCSs). The genomic architectures of the TCSs show that they fall into discrete numbers of classes. The genomic architecture is represented by the arrow diagram. The arrow depicts the genes, whereas the intergenic distances are represented by curly brackets. The direction of arrows in the TCS operon genes is a representation of those present in the positive strand. A similar arrangement is present in the negative strand. The length of the arrows is a representation of genes and not to scale. The TCSs in the other (general) category are shown to possess a higher number of discrete classes than the antibiotic resistance and virulence classes. (A) Genomic architecture of the PmrBA TCS involved in antibiotic resistance among the Gram-negative ESKAPEE pathogens K. pneumoniae, A. baumannii, P. aeruginosa, E. cloacae, and E. coli. (B) Genomic architecture of the WalKR TCS involved in virulence. The WalKR system is found in the Gram-positive ESKAPEE pathogens E. faecium and S. aureus. (C) Genomic architecture of the KdpDE potassium (K^+^)-sensing TCS in S. aureus, K. pneumoniae, A. baumannii, P. aeruginosa, E. cloacae, and E. coli.

We scanned the genomic architecture of the most frequently shared TCSs among ESKAPEE pathogens in the antibiotic resistance, virulence, and other (general) categories and found that it varies (Fig. 5 and Fig. S8). The main reason to plot the genomic architecture is to highlight the genomic arrangement of the TCSs among different organisms. As the same TCSs perform the same functions in different bacteria, we want to highlight the similarities/differences between the same TCSs among different bacteria. However, we also found some variation in gene arrangement within the same bacterial strains, e.g., the PmrBA, WalKR, and KdpDE TCSs, as shown in Fig. 5. Upon comparing the variations in gene arrangement in the TCS operons within each category, we found that more variation exists among TCSs in the other (general) category than in those involved in virulence and antibiotic resistance.

10.1128/mSystems.00981-20.8 FIG S8 Pangenome analysis of the two-component systems. (A) Genomic architecture of the BaeSR antibiotic resistance two-component system among Gram-negative ESKAPEE pathogens, i.e., K. pneumoniae, A. baumannii, E. cloacae, and E. coli. (B) Genomic architecture of the AgrCA virulence two-component system in E. faecium and S. aureus. (C) Genomic architecture of the VraSR antibiotic resistance two-component system. The VraSR system is found in Gram-positive ESKAPEE pathogens, i.e., E. faecium and S. aureus. (D) Genomic architecture of the AlgZR virulence two-component system among Gram-negative ESKAPEE pathogens, i.e., A. baumannii and P. aeruginosa. (E) Genomic architecture of the CusSR copper-sensing two-component system. The CusSR system is found in K. pneumoniae, A. baumannii, E. cloacae, and E. coli. Download FIG S8, PDF file, 0.5 MB. Copyright © 2021 Rajput et al. 2021 Rajput et al. This content is distributed under the terms of the Creative Commons Attribution 4.0 International license.

For example, the PmrBA two-component system has three genes in the operon: PmrB, PmrA, and PmrC. PmrBA is found in five Gram-negative ESKAPEE pathogens: E. coli, E. cloacae, P. aeruginosa, K. pneumoniae, and A. baumannii. The PmrBA operon shows different intergenic distances in these five pathogens despite them performing the same antibiotic resistance function. Likewise, the intergenic distances and gene arrangements vary among the bacteria with the WalKR and KdpDE two-component systems. For example, the WalKR operon is found in E. faecium and S. aureus, while KdpDE is found in S. aureus, K. pneumoniae, A. baumannii, P. aeruginosa, E. cloacae, and E. coli. Furthermore, we checked the correlation between the intergenic distances of the TCSs and the host range. We plotted the phylogenetic tree from the concatenated sequences of the TCS (WalKR) operon and compared it with the respective multilocus sequence typing (MLST) values (Fig. S9). The TCSs of S. aureus possess 2 different types of genomic architecture from 258 different MLST values, while the TCSs of E. faecium have 3 types of genomic rearrangement from 130 MLST profiles. From the correlation analysis, we found that the genomic architecture is not correlated with the MLST profiles.

10.1128/mSystems.00981-20.9 FIG S9 Phylogenetic tree showing the correlation between the genomic architecture of the WalKR two-component system of Enterococcus faecium and Staphylococcus aureus and the MLST values. The branch colors indicate different genomic architectures, and the node colors depict the MLST values. Download FIG S9, PDF file, 1.1 MB. Copyright © 2021 Rajput et al. 2021 Rajput et al. This content is distributed under the terms of the Creative Commons Attribution 4.0 International license.

### Sequence variation among the two-component systems.

The sequence and structural variations were checked in histidine kinase and response regulator components of the TCSs. The sequences of both the HKs and RRs were checked to discover the percent variation among them (Fig. 4C and Fig. S7A). For VraSR, VraS (HK) and VraR (RR) have variant scores of 0.27 and 0.18, respectively. In WalKR, the variant scores of WalK (HK) and WalR (RR) are 0.12 and 0.05, respectively. In general, the HK domain shows more variation than the RR. Among the HK domains, the N terminus shows more variability than the C terminus. This is further statistically validated by the skewness values of WalK and VraS of 0.27 and 0.27, respectively.

10.1128/mSystems.00981-20.7 FIG S7 Pangenome analysis of the two-component systems. (A) Sequence variant bar graphs of WalK and WalR TCSs. The graph is plotted as the percent variation versus the number of residues. (B) Principal-component analysis (PCA) curves showing histidine kinases and response regulators of Staphylococcus aureus and Acinetobacter baumannii. The PCA curves are plotted by using peptide features, i.e., amino acid composition, dipeptide composition, and tripeptide composition. Download FIG S7, PDF file, 0.6 MB. Copyright © 2021 Rajput et al. 2021 Rajput et al. This content is distributed under the terms of the Creative Commons Attribution 4.0 International license.

Additionally, the sequence variation of the RRs and HKs among ESKAPEE pathogens was checked and depicted in the form of three-dimensional (3D) principal-component analysis (PCA) plots. For example, the 3D PCA plots of S. aureus and A. baumannii are depicted in Fig. S7B. The RR sequences of the respective TCSs seem to be tightly clustered compared to the HK sequences. Taken together, the sequence variation analysis reflects that HK has more sequence variation than the RR in the ESKAPEE pathogens.

## DISCUSSION

In this study, we carried out a pangenome analysis of TCSs in ESKAPEE pathogens. The study was made possible due to the recent growth in the number of strain-specific sequences available for these pathogens. With respect to the phylogenetic distribution of TCSs, we find that the number of TCSs varies among ESKAPEE pathogens, and they are group specific, i.e., among Gram-positive and Gram-negative pathogens, except in the case of KdpDE. Most TCSs are conserved among the pathogens (found in the closed pangenome), except in the case of P. aeruginosa. With respect to sequence and structural variation, we find that TCS operons are stratified in discrete classes, which is more pronounced for TCSs involved in general functions. The histidine kinases that sense environmental signals show more variability than response regulators, which maintain cellular expression.

The ESKAPEE pathogens possess different categories of TCSs (see Data Set S1, sheet 1, in the supplemental material). The numbers and types of TCSs reflect the characteristics of the particular bacterium. For example, most of the TCSs in P. aeruginosa are related to biofilm formation, while in A. baumannii, they deal with metal sensing. We found that the majority of TCSs are shared among members of the two major bacterial groups (Gram-positive or Gram-negative bacteria), while fewer of them are exclusive to an individual ESKAPEE pathogen (19, 27). Pangenomic analysis of TCSs allows us to decipher their phylogenetic distribution and conservation.

The TCS pangenomes of most ESKAPEE pathogens are found to be closed, which adds to their value as potential conserved targets for a species (28). Furthermore, any mutation in some TCS genes leads to deleterious effects on cell survival due to the negative fitness Z-score. The pangenome analysis further shows that various TCSs are common to more than one ESKAPEE pathogen, including VraSR (antibiotic resistance); AlgZR (virulence); and CitAB, PhoRP, and UhpBA (others [general]). Thus, these TCSs could serve as candidates for broad-spectrum inhibitors (26). However, some TCSs were also part of the variant, or accessory, pangenome, which is present in a particular subset of strains.

The closed ESKAPEE TCS pangenomes reflect their conservation status and should make them good targets with regard to pathogenicity and antibiotic resistance. P. aeruginosa has the highest number of TCSs in the core component of the pangenome. Surprisingly, P. aeruginosa strain CLJ1 seems to be an outlier because it carries a total of 33 TCSs, 5 of which are unique to this strain (including BfmSR, CarSR, CprSR, MifSR, and RoxSR) and 8 of which are shared across <10% of P. aeruginosa strains (including BfiSR, CpxAR, CzcSR, PirSR, PmrBA, PprAB, RcsCB, and RocS2A2). CLJ1 was isolated in 2010 from the lungs of a patient with fatal hemorrhagic pneumonia in France and contains an elevated number of ISL3 family insertions affecting major virulence-associated phenotypes and increased antibiotic resistance (29). Previously, TCSs have been proven to be important drug candidates, which are more promising than other conventional drugs due to the fact that the TCSs are ubiquitous, and the HK and RR are well conserved and surrounded by active sites. The TCSs are integral components of adaptive regulatory processes and utilized by the pathogenic bacteria to sense their environments. The high degree of structural homology between the catalytic domains of the HK and the RR in bacteria suggests that multiple TCSs can be inhibited by a single compound (30–32). Therefore, these TCSs could be used to develop antibacterial drugs as they are absent in humans and inhibit the virulence of bacteria without the development of resistance (31). However, a few TCSs inhibitors, like walkmycin A, a few thiazolidinone derivatives, and autoinducing peptides, etc., have been described to affect the pathogenic bacteria but do not show promising effects due to their poor selectivity (32). In this regard, we analyzed gene essentiality via fitness score, distribution, conservation, and functionality, etc., to confirm the possibility of some TCSs as promising drug candidates.

While the shared TCSs among different bacterial species exhibit the same function, the genomic architecture differs. The intergenic distances within the genes in an operon are thought to be evolutionarily conserved among a broad range of prokaryotes (33). However, we found that the genomic arrangements of the TCS operons fall into discrete classes. In a previous study, the *agr* operon in S. aureus was shown to fall into discrete classes that correlated with the host range of a given strain (34). In this study, we show that the genomic architectures of TCS operons generally fall into discrete classes, which are more pronounced in the TCSs performing other (general) functions (Fig. 3). As mentioned above, the intergenic distances were considered a marker of phylogenetic relatedness. In our analysis, we did not find any correlation between the TCS architectures and the MLST values. Thus, this shows that the genomic arrangement of the TCSs is not determined solely by the evolutionary forces that determine the phylogroup, but some other selective pressures might be responsible for the differences in architecture (on top of neutral background substitution bias) rather than performing the same function.

Histidine kinases and response regulators comprise a TCS. The HK is membrane bound, while the RR is its cytoplasmic counterpart (9). HK genes are found to be more sequence variable than RR genes. The variation in the HK sequence is especially pronounced in its N-terminal domain, likely due to its function as a sensor for a broad range of environmental signals. Our results are in agreement with those of previous studies that showed that the N termini in HKs are responsible for signal sensing, while the cytoplasmic C termini help with phosphate transfer (35).

### Conclusion.

As antibiotic resistance represents a major health concern worldwide, there is a growing need to identify new and promising targets in pathogenic bacteria. This first comprehensive pangenomic study of TCSs confirms their conservation and universality among ESKAPEE pathogens. The TCSs with negative fitness Z-values as well as essential functions could be used as promising drug targets, e.g., BaeSR, KdpDE, EvgSA, RstBA, DcuSR, and TorSR, etc. Among these six TCSs, KdpDE and BaeSR have been used to develop drugs; however, the remaining four TCSs, i.e., EvgSA, RstBA, DcuSR, and TorSR, have not been used to develop drugs to date. Given that TCSs are integral mechanisms that enable antibiotic resistance, virulence, and basic metabolic functions, they could be targeted to tackle pathogenicity and reduce antibiotic resistance among nosocomial infections caused by ESKAPEE pathogens.

## MATERIALS AND METHODS

The overall methodology is provided in Fig. 1B and is described in detail below.

### Collection and quality control of ESKAPEE genomes.

The ESKAPEE genomes were downloaded from the Pathosystems Resource Integration Center (PATRIC) v3.5.43 database (36). The downloaded genome has “complete” and “draft” genome statuses, “human, Homo sapiens” host, and “good” genome quality. Furthermore, the five levels of quality control (QC) were done to get a more refined set of genomes for downstream analysis. First, the genomes annotated as “plasmid” were removed. Second, the genomes that did not have multilocus sequence typing (MLST) data were removed. MLST filtration is important to have only the genomes with the presence of housekeeping genes to provide a good resolution of genome characterization. Third, only those genomes with <100 contigs were retained, to confer a good-quality assembly. Fourth, genomes with the coding region of genes, i.e., coding DNA sequences (CDSs), between the [average ± 2(standard deviation)], were kept, to remove the misannotated genomes. Fifth, the genomes with >1,000 N’s were filtered out. Tables depicting the resulting ESKAPEE pathogen genomes at each quality control step are provided in Fig. S1 to S3 in the supplemental material.

10.1128/mSystems.00981-20.1 FIG S1 (A) Table showing the total number of genomes filtered at each quality control step. (B and C) Flow charts depicting the quality control steps and scatter plots for Enterococcus faecium (B) and Staphylococcus aureus (C). CDS is the coding DNA sequence, and N is an unidentified base. Download FIG S1, PDF file, 0.4 MB. Copyright © 2021 Rajput et al. 2021 Rajput et al. This content is distributed under the terms of the Creative Commons Attribution 4.0 International license.

10.1128/mSystems.00981-20.2 FIG S2 Flow charts depicting the quality control steps and scatter plots for Pseudomonas aeruginosa (A), Escherichia coli (B), and Klebsiella pneumoniae (C). CDS is the coding DNA sequence, and N is an unidentified base. Download FIG S2, PDF file, 0.5 MB. Copyright © 2021 Rajput et al. 2021 Rajput et al. This content is distributed under the terms of the Creative Commons Attribution 4.0 International license.

10.1128/mSystems.00981-20.3 FIG S3 Flow charts depicting the quality control steps and scatter plots for Enterobacter cloacae (A) and Acinetobacter baumannii (B). CDS is the coding DNA sequence, and N is an unidentified base. Download FIG S3, PDF file, 0.4 MB. Copyright © 2021 Rajput et al. 2021 Rajput et al. This content is distributed under the terms of the Creative Commons Attribution 4.0 International license.

### Annotation of two-component systems among the ESKAPEE pathogens.

The hidden Markov model (HMM) (37) and BLAST (38) were used to annotate the TCSs among all the ESKAPEE pathogens. The HMM profile information for the HKs and RRs were collected from MIST3.0 (39), P2CS (40), and the literature. The Pfam profiles of the RRs and HKs in all ESKAPEE pathogens were downloaded using Pfam32.0 (41). The Pfam profiles are the summarized outputs of protein sequences of the family and built through seed and automatically generated full alignments (42). Later on, hmmsearch was used to annotate the TCS proteins among ESKAPEE pathogens. This method is highly robust as we have used a threshold E value of 0.01 and a score of ≥0.25 to filter the hits from hmmsearch. A table showing the Pfam profiles used is depicted in Data Set S1, sheet 4.

### Summarizing the two-component systems among the ESKAPEE pathogens.

The annotated TCS proteins of ESKAPEE pathogens were curated and summarized. The summarization of TCSs was done broadly using four categories, i.e., antibiotic resistance, virulence, others/general, and predicted/unknown function. In the current study, we are focused on antibiotic research on the ESKAPEE pathogens, such as antibiotic resistance and virulence. Therefore, we put the remaining TCSs, such as biofilm, quorum sensing, pH, and motility, in the other (general) category. All the TCSs were scanned for their frequency of occurrence among the individual pathogens. Afterward, four heat maps were constructed for the above-mentioned categories with the information on the frequency of occurrence of the TCSs (HK and RR) among them.

### Pangenomic analysis of two-component systems among the ESKAPEE pathogens.

We performed a pangenomic analysis of all the TCS proteins by checking their distribution among strains. Furthermore, the frequency distributions of the TCSs in all or at least 98% strains (considered core), some strains (accessory), or only one strain (unique) were determined (43). The distribution was calculated as (strain with the presence of TCSs/overall strains) × 100.

For each species, we plotted proxy pangenome and core genome curves as described previously (44), but we limited our input to TCSs. Briefly, we generated 1,000 random permutations of the input genomes, and for each permutation, we randomly sampled strains one at a time without replacement. At the first draw, we counted the number of TCSs detected. At the next draw, we counted the number of TCSs but subdivided them into three counts: (i) the core count, i.e., the number of unique TCSs found in both draws; (ii) the pangenome count, i.e., the total number of unique TCSs when pooling the two draws; and (iii) the new TCS count, i.e., the number of TCSs found in the second draw that we could not find in the first draw. This process was repeated until all strains were drawn. We generated a vector of recorded set sizes for each of the 1,000 permutations and calculated the average and standard deviation for each step. We then fit Heaps’ law (an empirical power law) to the vector of new gene sets and calculated the means and standard deviations of the fitted parameters α and *k*. Heaps’ law was originally developed to describe the count of unique words in a text as a function of the length of the text. Here, it can be expressed as *n* = *k* × *N*^−α^, where *n* is the total count of new TCSs discovered at each draw, *N* is the total number of genomes, *k* is a multiplicative constant, and α is the gene discovery decay rate (45). The pangenome can be described as either “closed” (α > 1) or “open” (α < 1). A pangenome is open when the pan count increases indefinitely as new genomes are considered and closed when the rate of increase of the pan count slows down as more strains are analyzed and the pan count eventually reaches a plateau (at which point no new genes are discovered).

### Gene essentiality and fitness score.

The essential genes are indispensable for cell survival. The gene essentiality of the TCSs among the ESKAPEE pathogens is determined using the DEG (46) and OGEE (47) databases. Furthermore, the fitness score of the cell is determined by the BacFITBase database (48). A negative value of the fitness score of a gene shows that the removal of the gene impairs the cell function of the pathogen, while a positive fitness score means that the removal of the gene is not lethal but results in decreased fitness of the pathogen. A fitness Z-score of <0 indicates that a given mutation is more detrimental than the average mutation during infection and results in a negative effect on the pathogen. Among the ESKAPEE pathogens, the BacFITBase database contains the fitness scores of E. coli, A. baumannii, and K. pneumoniae.

### Sequence variation among two-component systems of the ESKAPEE pathogens.

The sequences of the RRs and HKs of the TCSs were used for analysis. Furthermore, BLASTp (38) was run between the sequences and the respective reference sequences. Any insertions, deletions, or single nucleotide polymorphisms (SNPs) between the RR or HK sequences and the reference sequence were counted as a variant residue at the residue position of the reference sequence. These were calculated by taking the total number of variants found in each protein by BLASTp (differences between the protein and the reference sequence for the protein) and dividing that number by the total number of proteins and then again by the length of the reference sequence. This is the average number of variants per amino acid of the original sequence.

We calculated the variants according to the formula number of amino acid variants/number of amino acids/total number of sequences. For example, say gene A is 200 amino acids (aa) long. We compare 100 sequences to it and find 50 total variant positions (50 aa that are different from the reference). The end variant score would be (50/100)/200 = 0.0025.

We also performed a statistical comparison, where we checked the skewness, i.e., how far the data are skewed from the uniform distribution. If the skewness value is >0, there is more weight in the left tail of the distribution, and if the skewness value is <0, there is more weight in the right tail of the distribution.

The sequence variations among the RR and HK sequences were also determined using principal-component analysis (PCA) plots. As we want to explore the peptide sequences, the use of the best descriptive features is important. For this, the best and simplest descriptive features are amino acid composition, dipeptide composition, and tripeptide composition, as used previously (49–51). Furthermore, important peptide features like amino acid composition, dipeptide composition, and tripeptide composition were calculated (49). Furthermore, these features were used to make PCA plots for RRs and HKs in all ESKAPEE pathogens.

### Genomic architectures of two-component systems among the ESKAPEE pathogens.

The genomic architecture provides an important idea about the spatial arrangement of the genes in an operon (34). Here, we constructed the genomic architectures of the most shared and important TCSs among categories such as antibiotic resistance, virulence, and others/general, for example, PmrAB, VraSR, and BaeSR (antibiotic resistance); AgrCA, WalKR, and AlgZR (virulence); and CusSR and KdpDE (others [general]) TCSs. The genome architecture was constructed using gene sequences of the TCSs and calculating the intergenic distances and orientations among them. All this information was collated and depicted in the form of arrow diagrams. Furthermore, we plotted phylogenetic trees of the TCSs and compared them with their respective MLST values. The MLST values represent a set of housekeeping genes in the bacteria and thus categorize a strain according to its unique allelic profile. The phylogenetic tree was plotted using concatenated TCS operon protein sequences in a maximum likelihood tree with 1,000 pseudoreplicates.

### Data availability.

The code used for the analysis of the study is available at https://github.com/akanksha-r/TCS_Pangenome.
