# Supplementary material for: Pangenome Analytics Reveal Two-Component Systems as Conserved Targets in ESKAPEE Pathogens
Source: mSystems. 2021 Jan 26;6(1):e00981-20. doi: 10.1128/mSystems.00981-20 (PMC7842365; doi:10.1128/mSystems.00981-20)
Supplement: FIG S4 [file mSystems.00981-20_sf004.pdf]

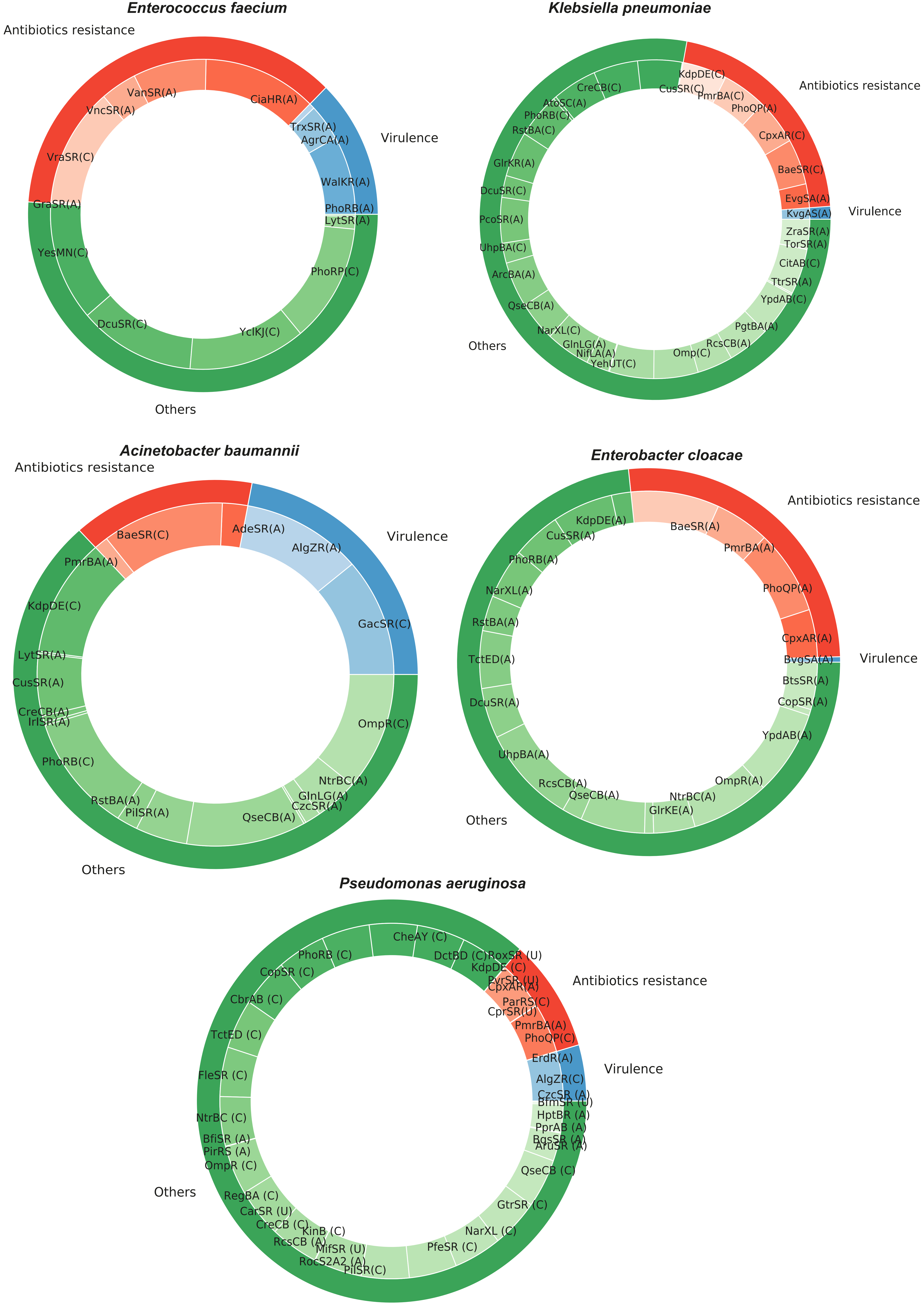
Enterococcus faecium
Antibiotics resistance
Virulence
Others
VanSR(A)
CiaHR(A)
VncSR(A)
TrxSR(A)
AgrCA(A)
VraSR(C)
WalKR(A)
PhoRB(A)
GraSR(A)
LytSR(A)
YesMN(C)
PhoRP(C)
DcuSR(C)
YclKJ(C)
Klebsiella pneumoniae
Antibiotics resistance
Virulence
Others
KdpDE(C)
CreCB(C)
CusSR(C)
AtoSC(A)
PmrBA(C)
PhoRB(C)
PhoQP(A)
RstBA(C)
CpxAR(C)
GlrKR(A)
BaeSR(C)
DcuSR(C)
EvgSA(A)
KvgAS(A)
PcoSR(A)
ZraSR(A)
UhpBA(C)
TorSR(A)
CitAB(C)
ArcBA(A)
TtrSR(A)
YpdAB(C)
QseCB(A)
PgtBA(A)
NarXL(C)
RcsCB(A)
GlnLG(A)
NifLA(A)
Omp(C)
YehUT(C)
Acinetobacter baumannii
Antibiotics resistance
Virulence
Others
BaeSR(C)
AdeSR(A)
AlgZR(A)
PmrBA(A)
KdpDE(C)
GacSR(C)
LytSR(A)
CusSR(A)
CreCB(A)
IrlSR(A)
OmpR(C)
PhoRB(C)
NtrBC(A)
GlnLG(A)
RstBA(A)
CzcSR(A)
PilSR(A)
QseCB(A)
Enterobacter cloacae
Antibiotics resistance
Virulence
Others
KdpDE(A)
BaeSR(A)
CusSR(A)
PmrBA(A)
PhoRB(A)
NarXL(A)
PhoQP(A)
RstBA(A)
CpxAR(A)
TctED(A)
BvgSA(A)
BtsSR(A)
CopSR(A)
DcuSR(A)
YpdAB(A)
UhpBA(A)
OmpR(A)
RcsCB(A)
QseCB(A)
NtrBC(A)
GlrKE(A)
Pseudomonas aeruginosa
Antibiotics resistance
Virulence
Others
CheAY (C)
PhoRB (C)
DctBD (C)
RoxSR (U)
KdpDE (C)
CopSR (C)
PvrSR (U)
CpxAR(A)
CbrAB (C)
ParRS(C)
CprSR(U)
TctED (C)
PmrBA(A)
PhoQP(C)
ErdR(A)
FleSR (C)
AlgZR(C)
CzcSR (A)
BfmSR (U)
NtrBC (C)
HptBR (A)
PprAB (A)
BfiSR (A)
BqsSR (A)
PirRS (A)
AruSR (A)
OmpR (C)
QseCB (C)
RegBA (C)
GtrSR (C)
CarSR (U)
CreCB (C)
NarXL (C)
KinB (C)
RcsCB (A)
PfeSR (C)
MifSR (U)
RocS2A2 (A)
PilSR(C)
