# Supplementary material for: Pangenome Analytics Reveal Two-Component Systems as Conserved Targets in ESKAPEE Pathogens
Source: mSystems. 2021 Jan 26;6(1):e00981-20. doi: 10.1128/mSystems.00981-20 (PMC7842365; doi:10.1128/mSystems.00981-20)
Supplement: FIG S5 [file mSystems.00981-20_sf005.pdf]

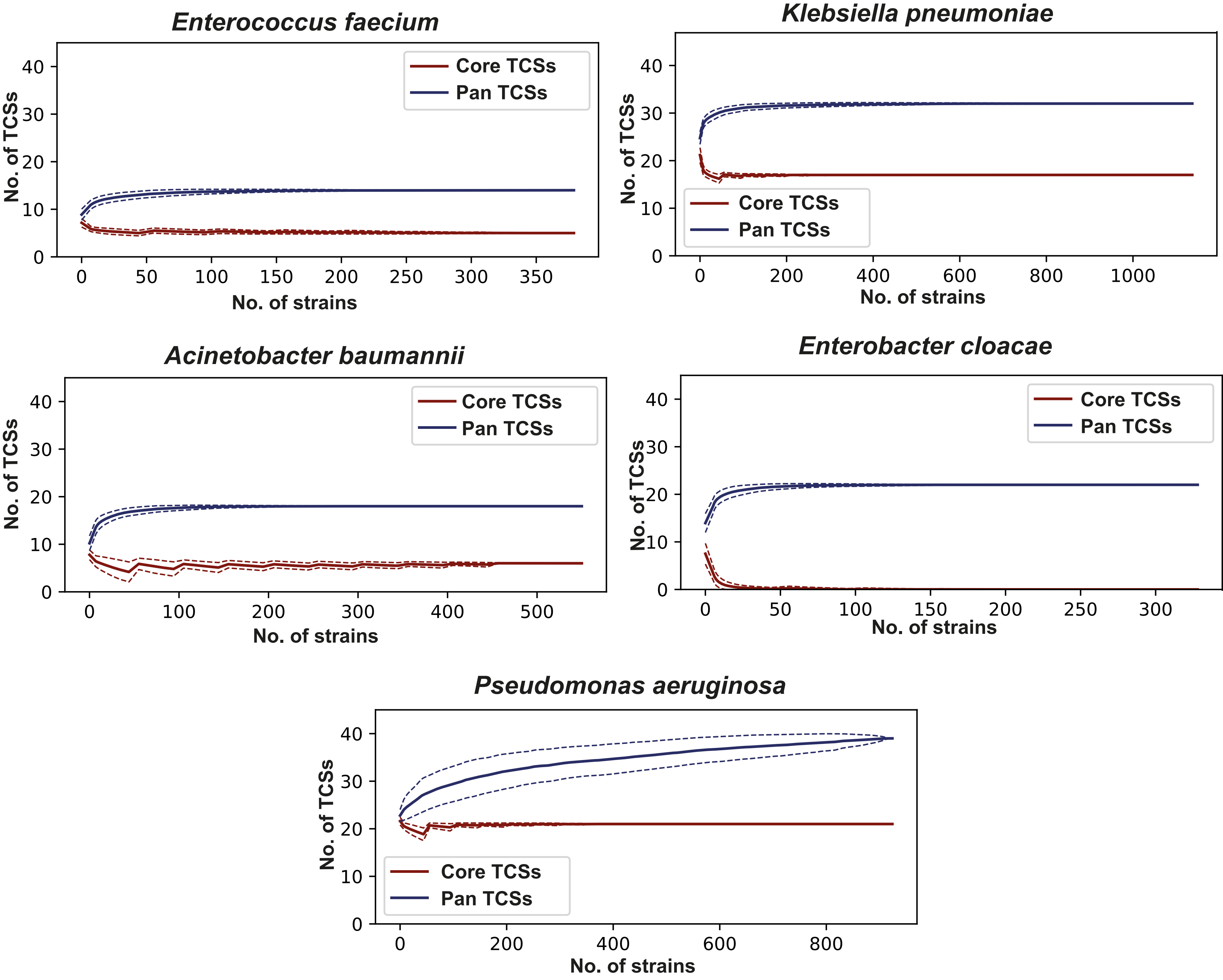
Enterococcus faecium
Core TCSs
Pan TCSs
No. of TCSs
0
10
20
30
40
0
50
100
150
200
250
300
350
No. of strains
Klebsiella pneumoniae
Core TCSs
Pan TCSs
No. of TCSs
0
10
20
30
40
0
200
400
600
800
1000
No. of strains
Acinetobacter baumannii
Core TCSs
Pan TCSs
No. of TCSs
0
10
20
30
40
0
100
200
300
400
500
No. of strains
Enterobacter cloacae
Core TCSs
Pan TCSs
No. of TCSs
0
10
20
30
40
0
50
100
150
200
250
300
No. of strains
Pseudomonas aeruginosa
Core TCSs
Pan TCSs
No. of TCSs
0
10
20
30
40
0
200
400
600
800
No. of strains
