# Supplementary figures and images for: Pangenome Analytics Reveal Two-Component Systems as Conserved Targets in ESKAPEE Pathogens
Source: mSystems. 2021 Jan 26;6(1):e00981-20. doi: 10.1128/mSystems.00981-20 (PMC7842365; doi:10.1128/mSystems.00981-20)

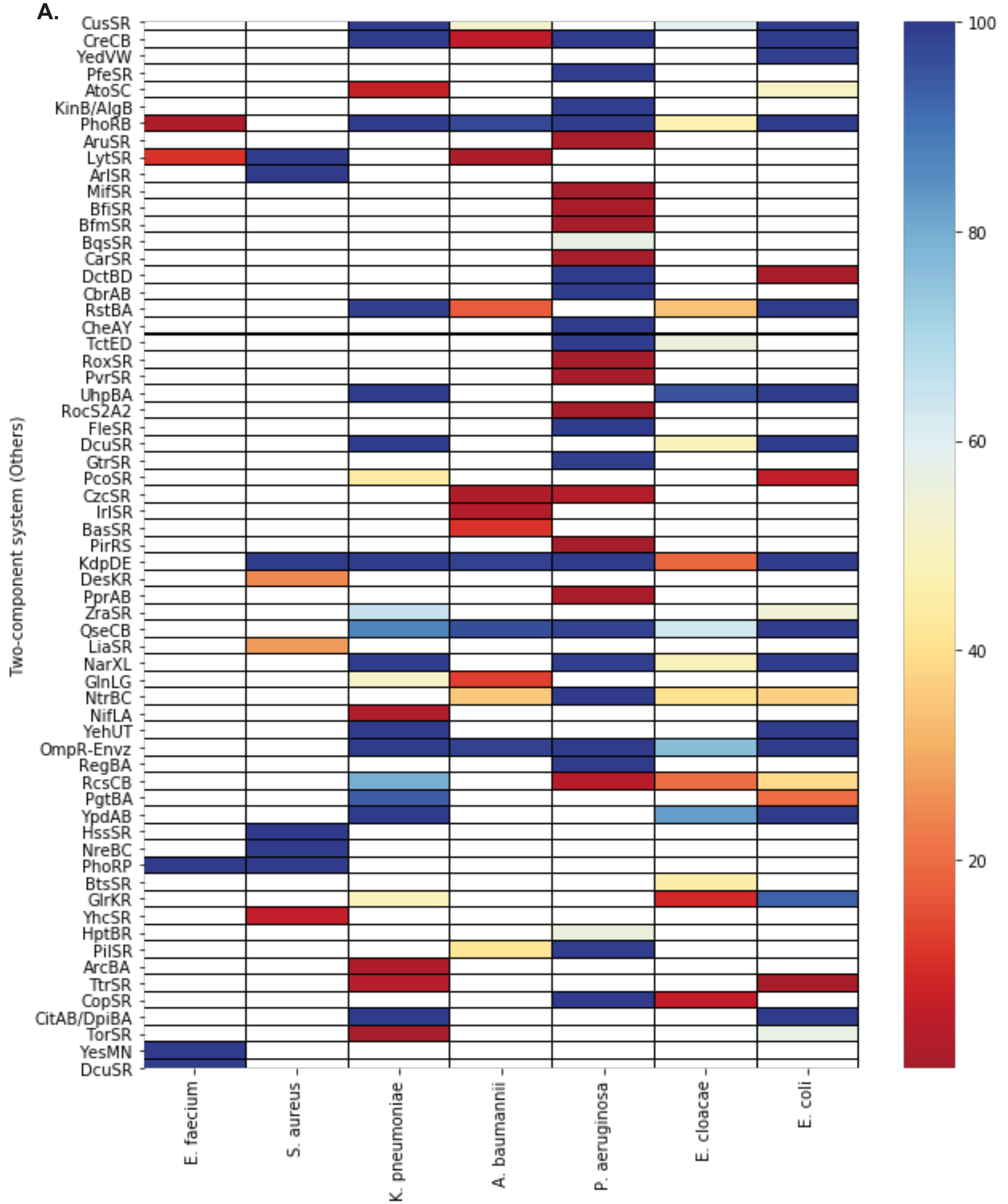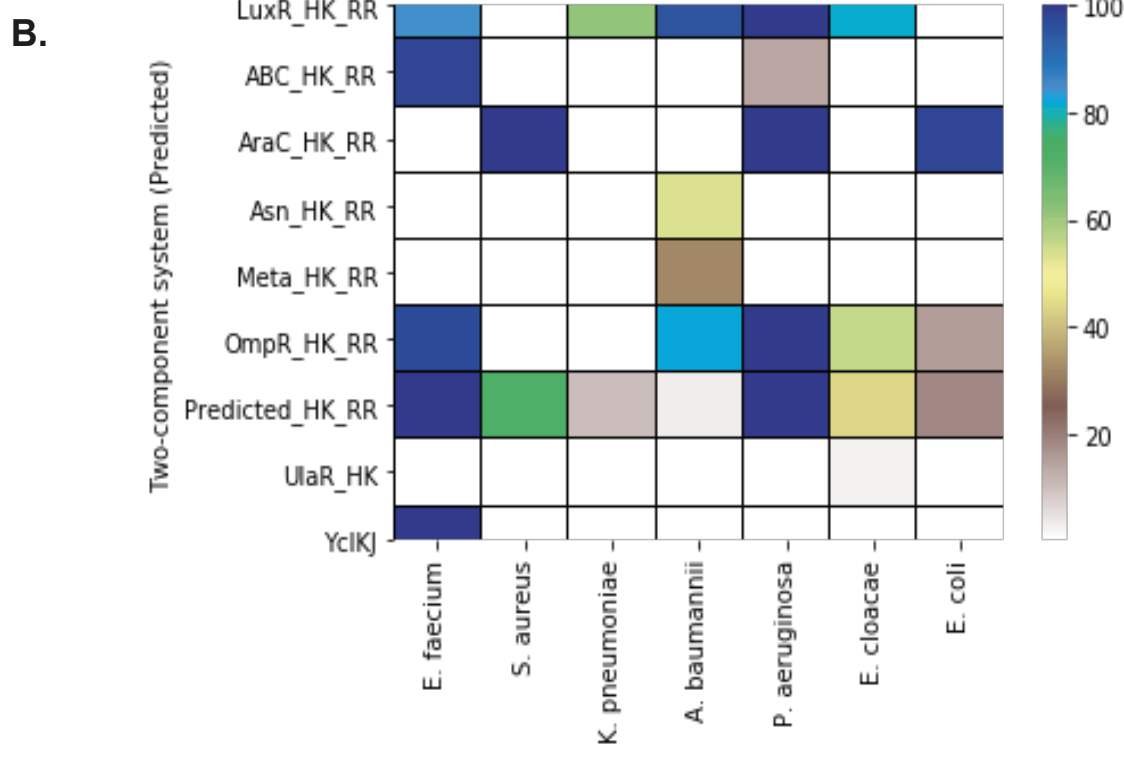

Supplement: FIG S6 [file mSystems.00981-20_sf006.pdf]

**A. *E. faecium***

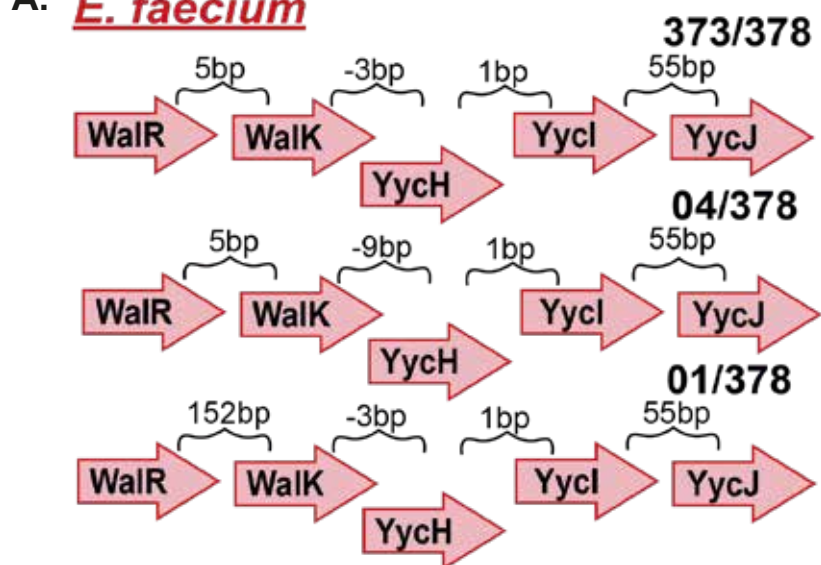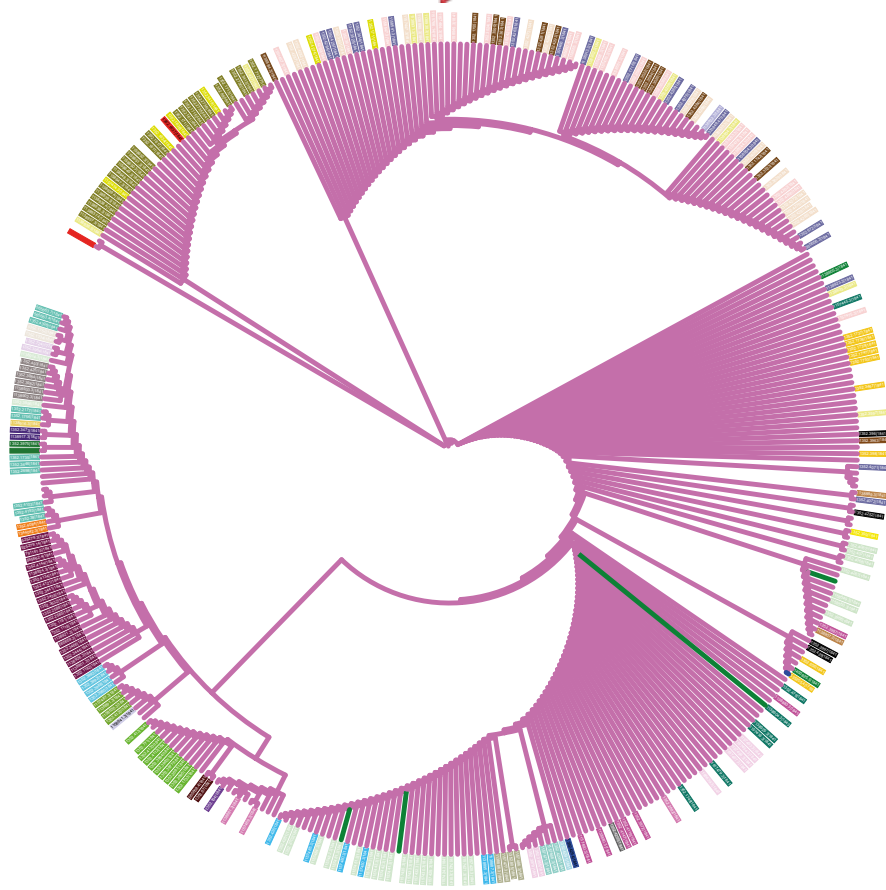

**B. *S. aureus***

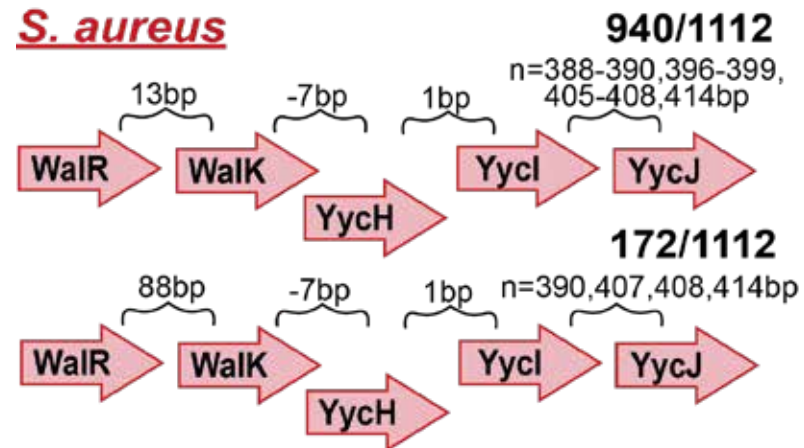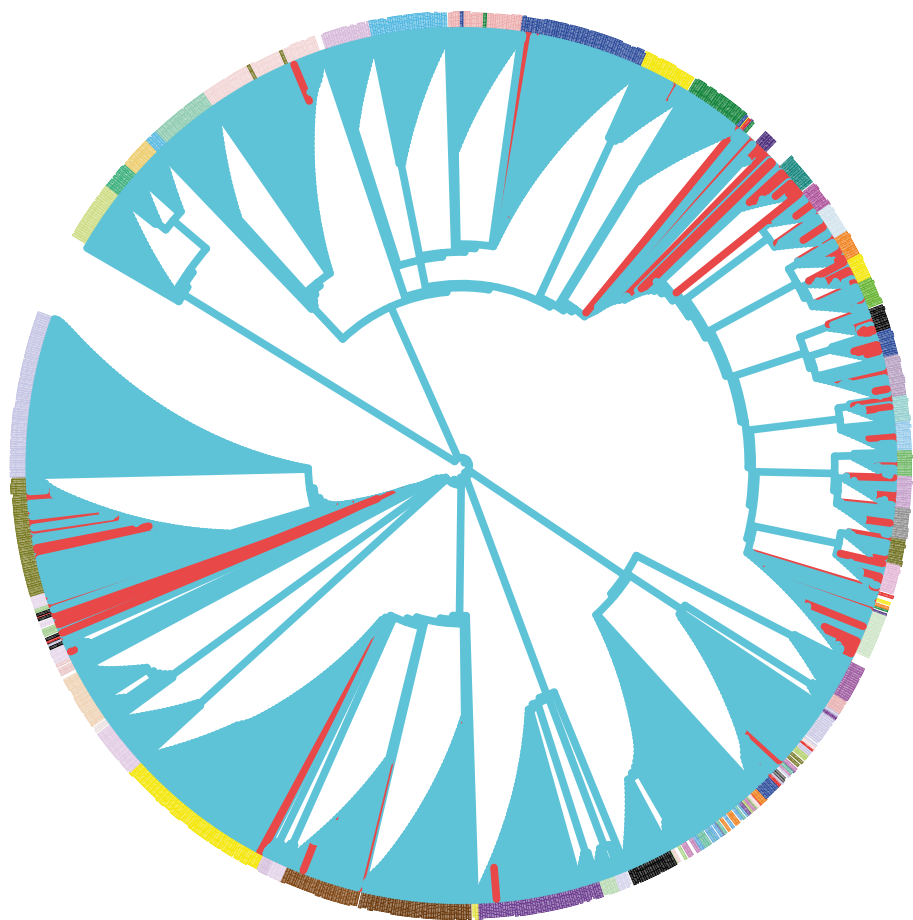

Supplement: FIG S9 [file mSystems.00981-20_sf009.pdf]
