# Supplementary material for: Pangenome Analytics Reveal Two-Component Systems as Conserved Targets in ESKAPEE Pathogens
Source: mSystems. 2021 Jan 26;6(1):e00981-20. doi: 10.1128/mSystems.00981-20 (PMC7842365; doi:10.1128/mSystems.00981-20)
Supplement: FIG S8 [file mSystems.00981-20_sf008.pdf]

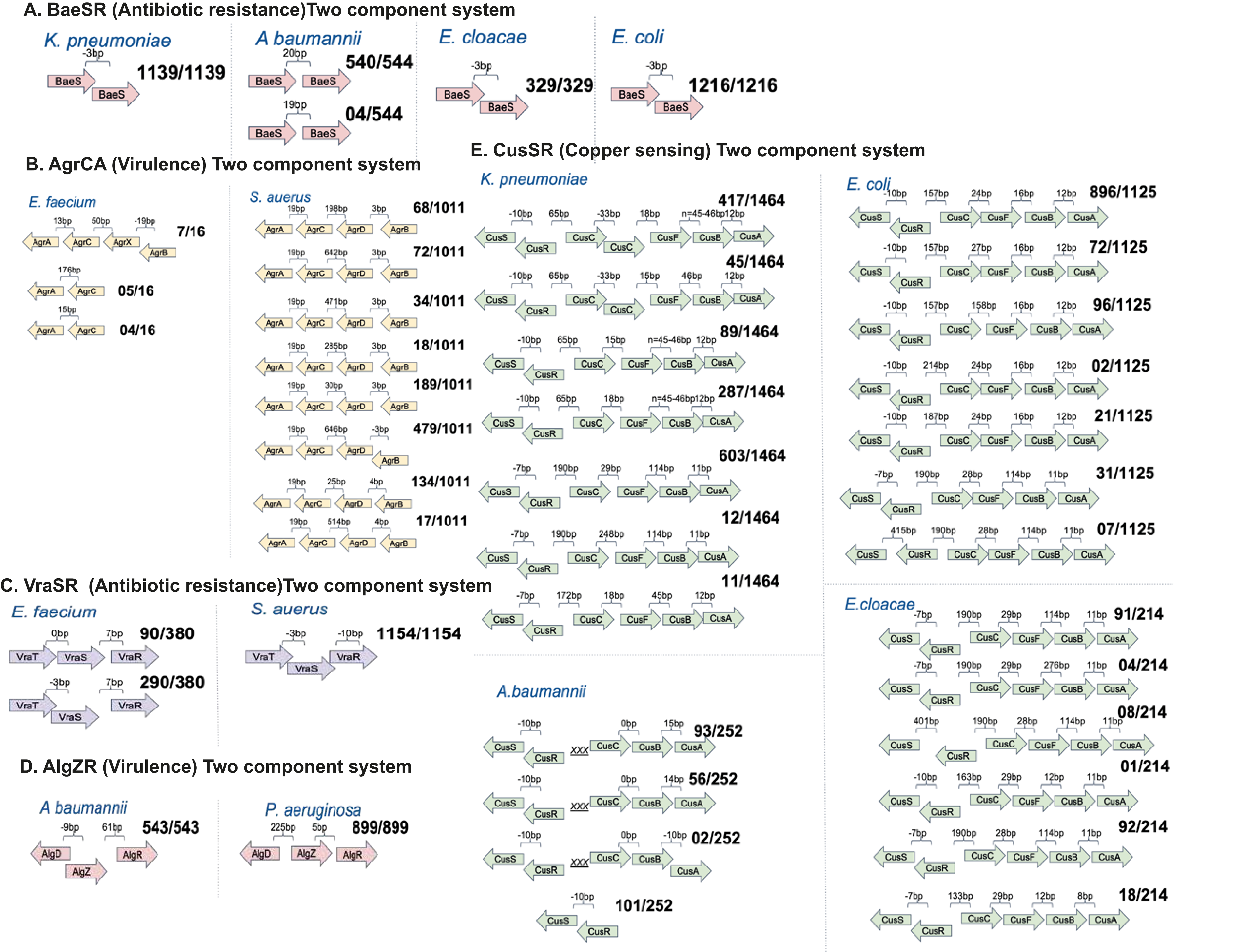
A. BaeSR (Antibiotic resistance)Two component system
K. pneumoniae
-3bp
BaeS
BaeS
1139/1139
A baumannii
20bp
540/544
19bp
04/544
E. cloacae
-3bp
329/329
E. coli
-3bp
1216/1216
B. AgrCA (Virulence) Two component system
E. faecium
13bp
50bp
-19bp
AgrA
AgrC
AgrX
AgrB
7/16
176bp
05/16
15bp
04/16
S. auerus
68/1011
72/1011
34/1011
18/1011
189/1011
479/1011
134/1011
17/1011
AgrD
C. VraSR (Antibiotic resistance)Two component system
E. faecium
0bp
7bp
VraT
VraS
VraR
90/380
-3bp
290/380
S. auerus
-3bp
-10bp
1154/1154
D. AlgZR (Virulence) Two component system
A baumannii
-9bp
61bp
AlgD
AlgZ
AlgR
543/543
P. aeruginosa
225bp
5bp
899/899
E. CusSR (Copper sensing) Two component system
K. pneumoniae
CusS
CusR
CusC
CusF
CusB
CusA
417/1464
45/1464
89/1464
287/1464
603/1464
12/1464
11/1464
E. coli
896/1125
72/1125
96/1125
02/1125
21/1125
31/1125
07/1125
A.baumannii
xxx
93/252
56/252
02/252
101/252
E.cloacae
91/214
04/214
08/214
01/214
92/214
18/214
