# Supplementary material for: Pangenome Analytics Reveal Two-Component Systems as Conserved Targets in ESKAPEE Pathogens
Source: mSystems. 2021 Jan 26;6(1):e00981-20. doi: 10.1128/mSystems.00981-20 (PMC7842365; doi:10.1128/mSystems.00981-20)

A.

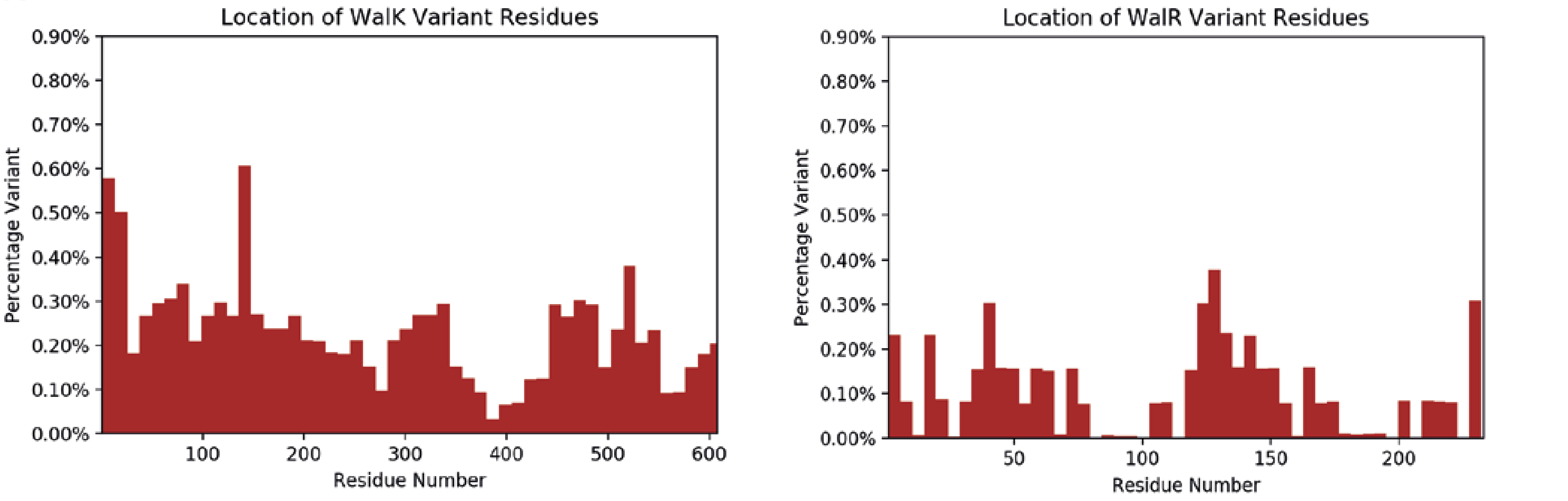

PCA for Two Component systems of HK in Staphylococcus aureus

PCA for Two Component systems of RR in Staphylococcus aureus

B.

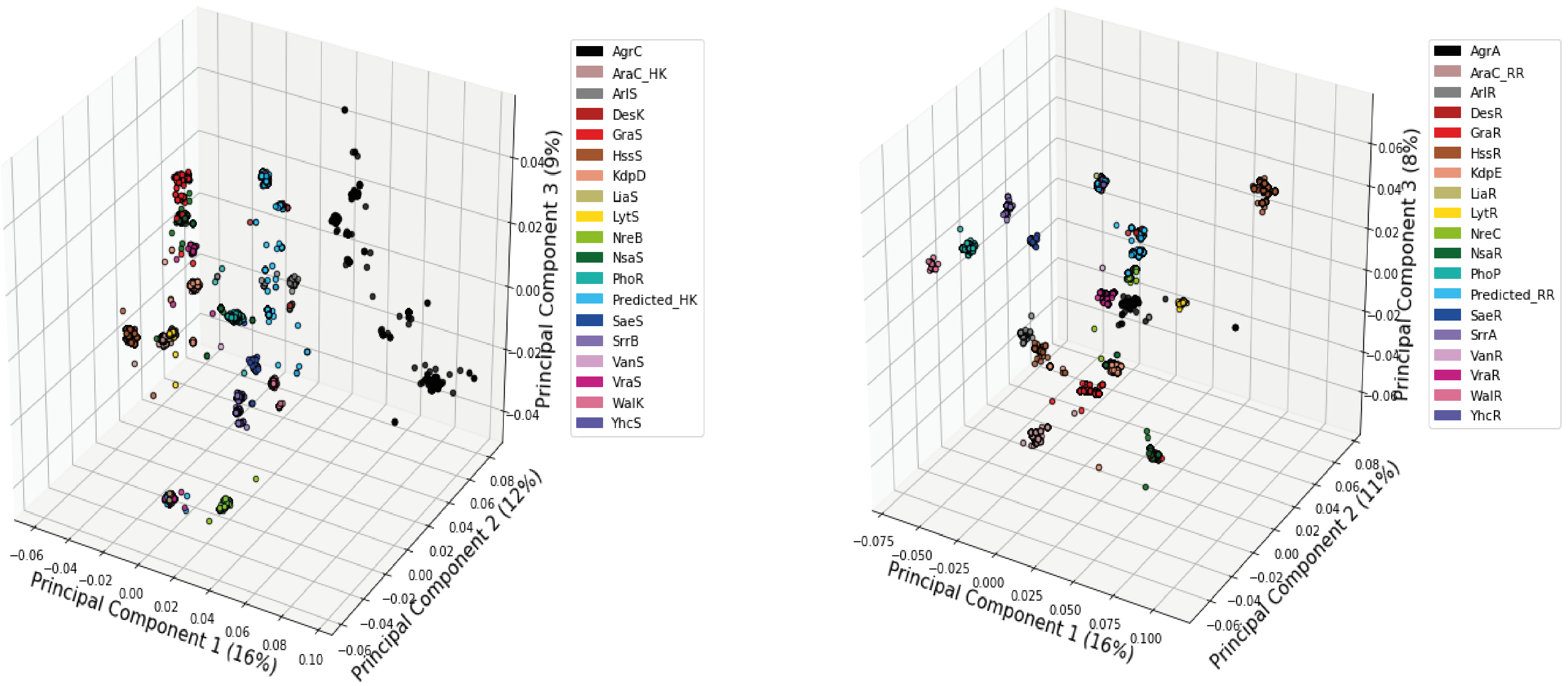

PCA for Two Component systems of HK in Acinetobacter baumannii

PCA for Two Component systems of RR in Acinetobacter baumannii

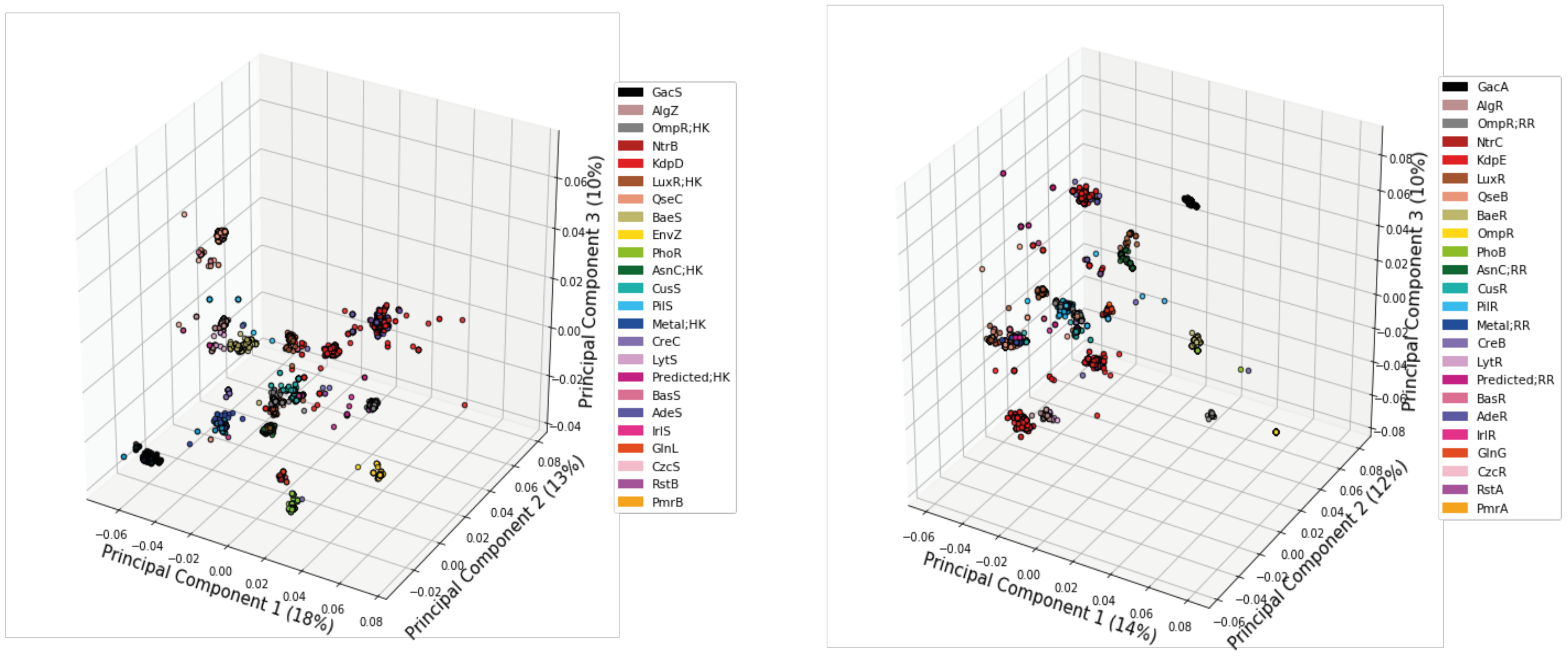

Supplement: FIG S7 [file mSystems.00981-20_sf007.pdf]
