# Supplementary material for: Pangenome Analytics Reveal Two-Component Systems as Conserved Targets in ESKAPEE Pathogens
Source: mSystems. 2021 Jan 26;6(1):e00981-20. doi: 10.1128/mSystems.00981-20 (PMC7842365; doi:10.1128/mSystems.00981-20)

A.

Pathogens	Genomes	QCQA1	QCQA2	QCQA3	QCQA4	QCQA5
Enterococcus faecium	1586	1417	1212	390	381	381
Staphylococcus faecium	8132	8132	1208	1208	1166	1166
Klebsiella pneumoniae	7073	7073	2106	1189	1141	1141
Acinetobacter baumannii	3666	2648	1135	577	556	556
Pseudomonas aeruginosa	2256	2256	1771	969	929	929
Enterobacter cloacae	815	815	485	342	330	330
Escherichia coli	8554	8554	3711	1271	1226	1226

B. *Enterococcus faecium*

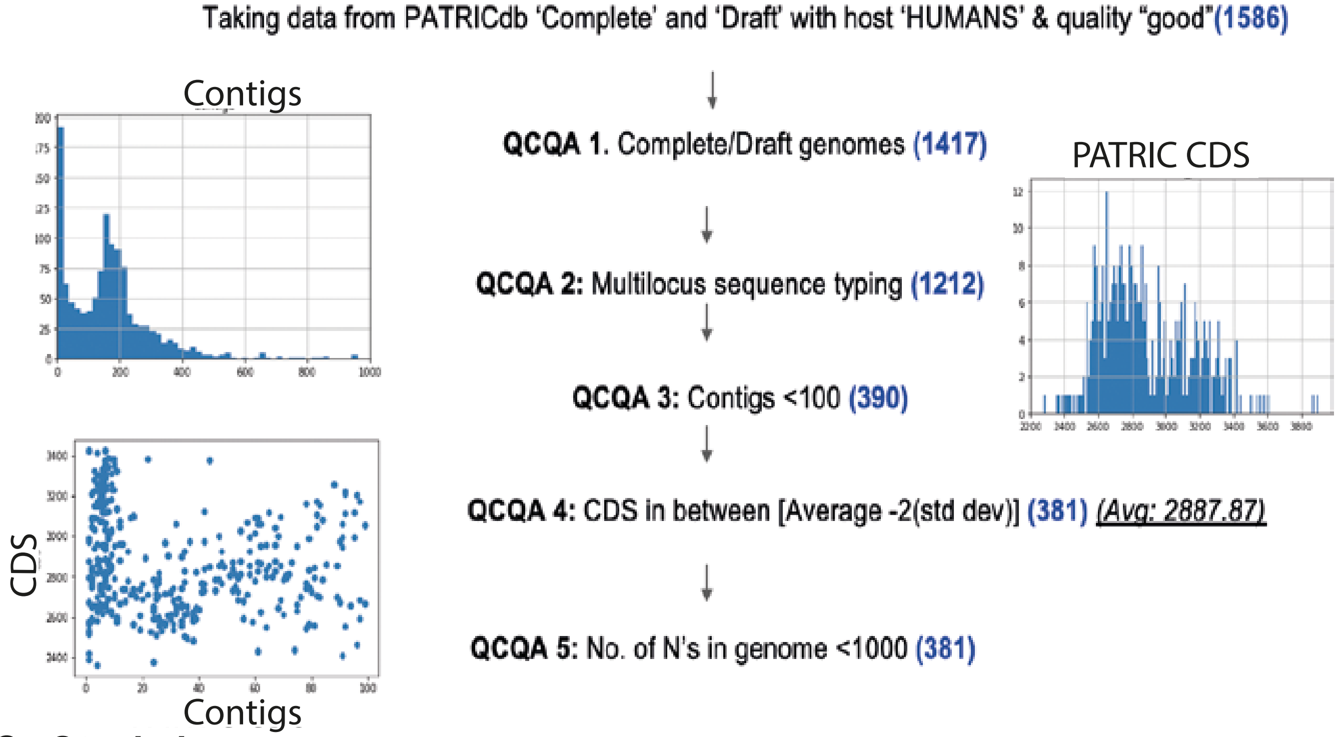

C. *Staphylococcus aureus*

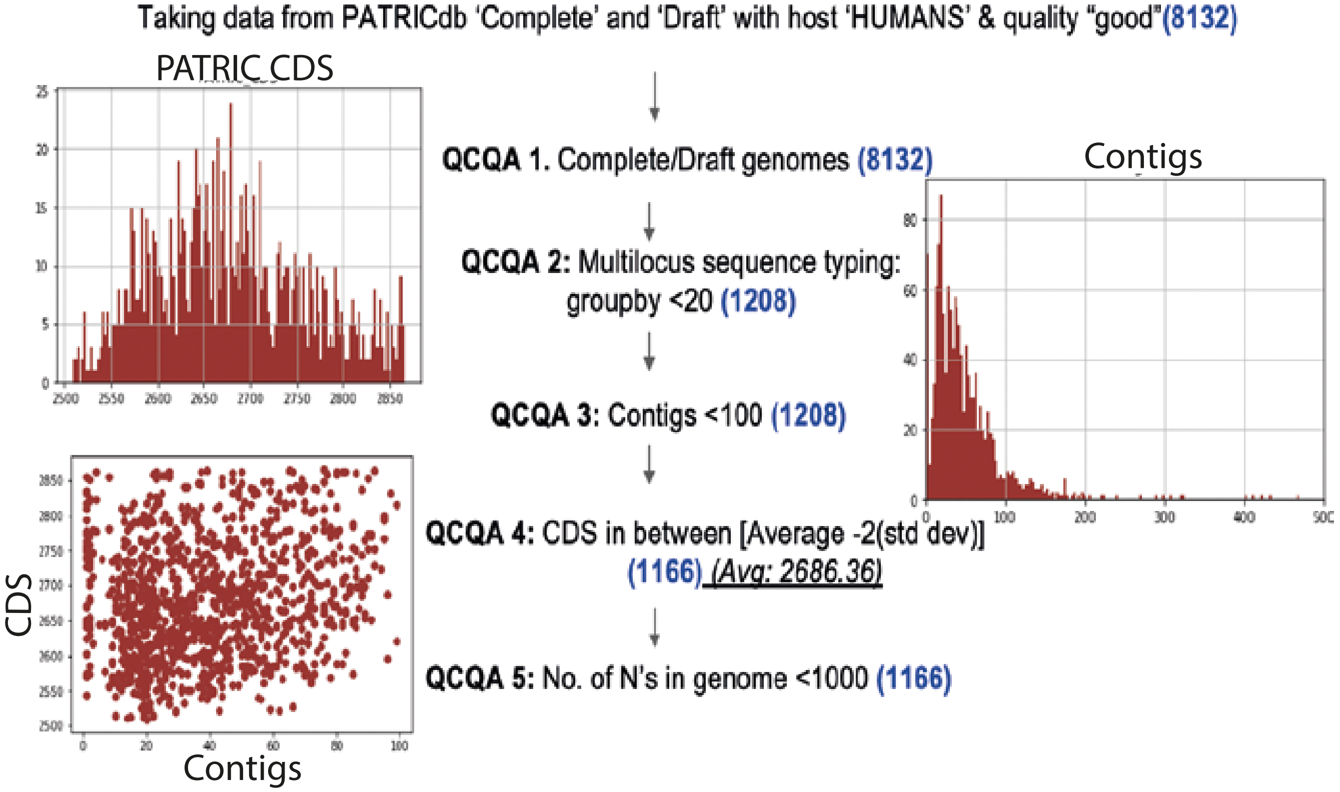

Supplement: FIG S1 [file mSystems.00981-20_sf001.pdf]
