# Supplementary material for: Pangenome Analytics Reveal Two-Component Systems as Conserved Targets in ESKAPEE Pathogens
Source: mSystems. 2021 Jan 26;6(1):e00981-20. doi: 10.1128/mSystems.00981-20 (PMC7842365; doi:10.1128/mSystems.00981-20)
Supplement: FIG S2 [file mSystems.00981-20_sf002.pdf]

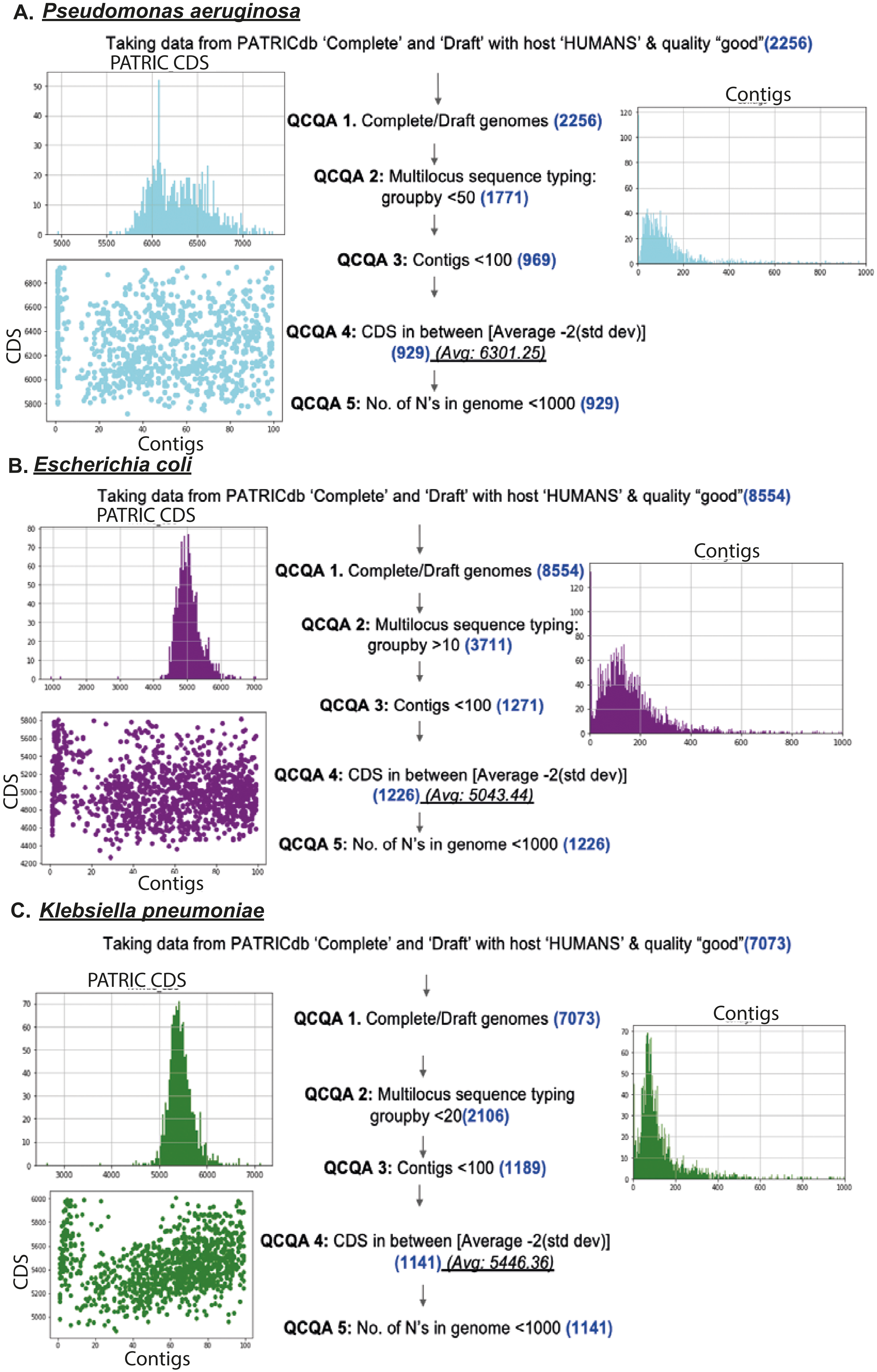

A. Pseudomonas aeruginosa
Taking data from PATRICdb 'Complete' and 'Draft' with host 'HUMANS' & quality "good"(2256)
PATRIC CDS
Contigs
QCQA 1. Complete/Draft genomes (2256)
QCQA 2: Multilocus sequence typing: groupby <50 (1771)
QCQA 3: Contigs <100 (969)
QCQA 4: CDS in between [Average -2(std dev)] (929) (Avg: 6301.25)
QCQA 5: No. of N's in genome <1000 (929)
CDS
Contigs
B. Escherichia coli
Taking data from PATRICdb 'Complete' and 'Draft' with host 'HUMANS' & quality "good"(8554)
PATRIC CDS
Contigs
QCQA 1. Complete/Draft genomes (8554)
QCQA 2: Multilocus sequence typing: groupby >10 (3711)
QCQA 3: Contigs <100 (1271)
QCQA 4: CDS in between [Average -2(std dev)] (1226) (Avg: 5043.44)
QCQA 5: No. of N's in genome <1000 (1226)
CDS
Contigs
C. Klebsiella pneumoniae
Taking data from PATRICdb 'Complete' and 'Draft' with host 'HUMANS' & quality "good"(7073)
PATRIC CDS
Contigs
QCQA 1. Complete/Draft genomes (7073)
QCQA 2: Multilocus sequence typing groupby <20(2106)
QCQA 3: Contigs <100 (1189)
QCQA 4: CDS in between [Average -2(std dev)] (1141) (Avg: 5446.36)
QCQA 5: No. of N's in genome <1000 (1141)
CDS
Contigs
