# Supplementary material for: Pangenome Analytics Reveal Two-Component Systems as Conserved Targets in ESKAPEE Pathogens
Source: mSystems. 2021 Jan 26;6(1):e00981-20. doi: 10.1128/mSystems.00981-20 (PMC7842365; doi:10.1128/mSystems.00981-20)

A. *Enterobacter cloacae*

Taking data from PATRICdb 'Complete' and 'Draft' with host 'HUMANS' & quality "good"**(815)**

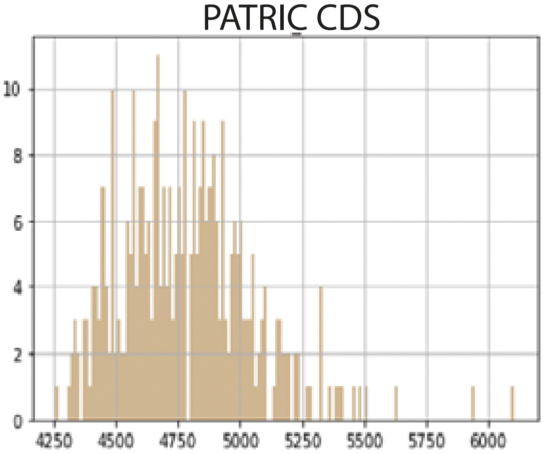

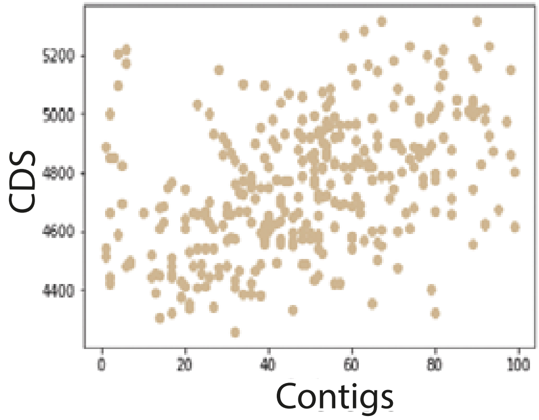

↓

QCQA 1. Complete/Draft genomes **(815)**

↓

QCQA 2: Multilocus sequence typing **(485)**

↓

QCQA 3: Contigs <100 **(342)**

↓

QCQA 4: CDS in between [Average -2(std dev)]
(330) *(Avg: 4783.83)*

↓

QCQA 5: No. of N's in genome <1000 **(330)**

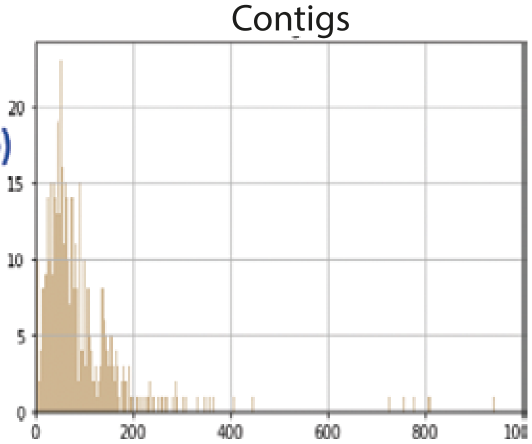

A. *Acinetobacter baumannii*

Taking data from PATRICdb 'Complete' and 'Draft' with host 'HUMANS' & quality "good"**(3666)**

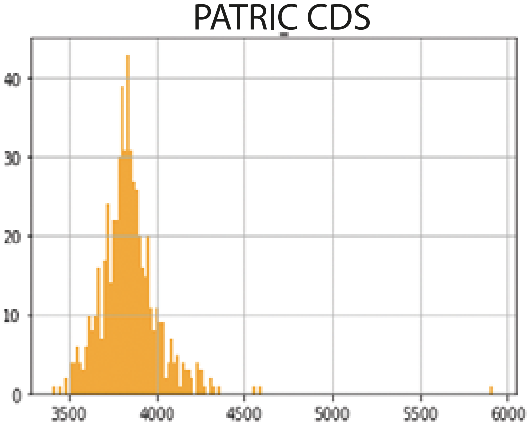

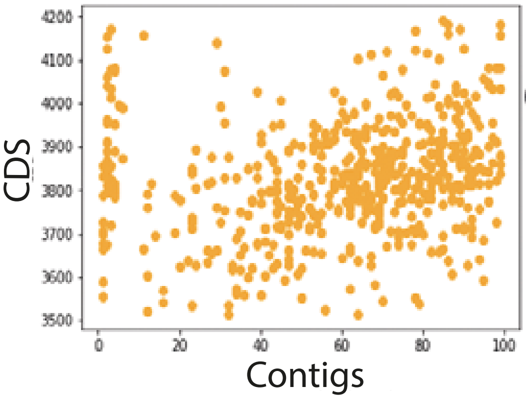

↓

QCQA 1. Complete/Draft genomes **(2648)**

↓

QCQA 2: Multilocus sequence typing
groupby >20 **(1135)**

↓

QCQA 3: Contigs <100 **(577)**

↓

QCQA 4: CDS in between [Average -2(std dev)]
(556) *(Avg: 3843.13)*

↓

QCQA 5: No. of N's in genome <1000 **(556)**

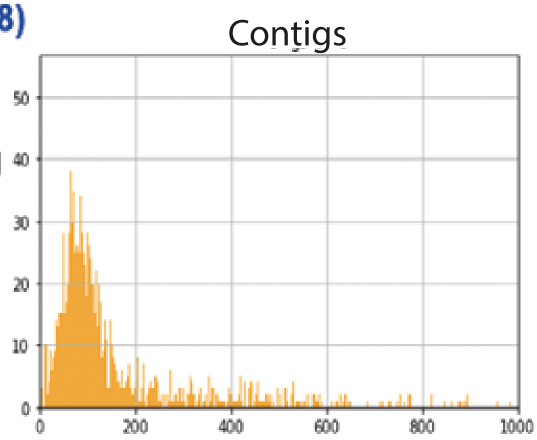

Supplement: FIG S3 [file mSystems.00981-20_sf003.pdf]
